# Tailored Organogel Systems for Optimized Pesticide Delivery: Mechanistic Insights and Agricultural Applications

**DOI:** 10.1002/advs.202519352

**Published:** 2026-01-28

**Authors:** Yue Wang, SiYu Xv, Chao Wu, Nan Li, GuoPeng Teng, XuePing Huang, Jian Luo

**Affiliations:** ^1^ School of Plant Protection Anhui Agricultural University Hefei Anhui P. R. China; ^2^ School of Chemistry and Materials Science University of Science and Technology of China Hefei Anhui P. R. China; ^3^ Institute of Plant Protection and Agro‐Product Safety Anhui Academy of Agricultural Sciences Hefei Anhui P. R. China

**Keywords:** foliar affinity, molecular dynamics, pesticide‐loaded organogels, rigid‐flexible morphological regulation, selective toxicity

## Abstract

Pesticide abuse poses a serious threat to food security and public health, while the scientific design of delivery systems is an important strategy for improving the precision of pesticide application. This study reports the development of a pesticide delivery platform using organogels to enhance stability in pest control and improve the simplicity and versatility of pesticide loading processes. The results show that the formation of the gel network occurs simultaneously with pesticide loading, achieving efficient pesticide capture at the molecular level. Meanwhile, the molecular configuration of the network can be flexibly tuned to adjust the mechanical strength of pesticide‐loaded organogels (PLOs), thereby enhancing their adaptability to biological interfaces. By constructing pesticide‐loaded organogel formulations (PLOFs) within micro‐scale reaction spaces, the system exhibits high topological affinity and controlled release behavior at biological interfaces in crop protection scenarios. Moreover, PLOFs demonstrate greater stability under climatic stress compared with traditional formulations. The study systematically elucidates the binding and release mechanisms between PLOs networks and pesticide molecules, and evaluates the efficacy and stability of PLOFs in various crop protection applications. This work highlights the great potential of PLOFs in enhancing pesticide utilization and provides a new perspective for the design of functional pesticide delivery systems.

## Graphical abstract

Utilizing molecular engineering for network design, three pesticide‐loaded organogels (PLOs) carriers exhibiting varying degrees of flexibility were prepared. The mechanical properties of these carriers, tunable via their network molecular structures, enhance their structural adaptability and retention stability at biological interfaces, thereby improving pesticide utilization efficiency under adverse climatic conditions. The flexible carrier, in particular, demonstrates flexible deformation characteristics, leading to improved delivery selectivity in complex crop protection scenarios, balancing targeted efficacy with non‐target safety.

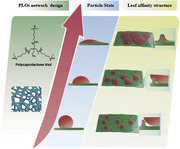

## 1 Introduction

The rational use of pesticides is crucial for food security and the sustainable development of the ecological environment [1, 2, 3]. With the development of materials science, the emergence of functionalized pesticide delivery systems (FPDSs) has provided a breakthrough technological approach for the efficient and targeted delivery of pesticides [4, 5, 6, 7]. FPDSs can precisely control the exposure and steady‐state retention levels of pesticides during application, achieve reduced dosage with increased efficacy, and effectively reduce environmental pollution, demonstrating great application potential in the field of plant protection [8, 9, 10, 11]. However, current FPDSs typically possess a complicated preparation process and low pesticides loading efficiency, since them require a two‐step process involving the synthesis of the carrier followed by pesticide loading [12, 13]. Compared with traditional pesticide microencapsulation techniques, the widespread applications of FPDSs in plant protection are severely hindered due to their challenges in reducing material costs and simplifying preparation processes [14]. Thus, a new FPDS model with simple preparation process and high efficient pesticide loading is urgently needed to be developed. To effectively optimize the preparation process and pesticide loading efficiency of FPDSs, integrating the synthesis of materials with the encapsulation of pesticides during one step within a single hydrophobic organic phase is a promising avenue [15].

Compared to the conventional “two‐step” method commonly adopted in FPDSs—pre‐synthesizing the carrier followed by separate loading—this one‐step loading strategy significantly simplifies the preparation process, effectively reduces procedural complexity, and substantially improves pesticide loading efficiency. Organogels, a typical hydrophobic systems, are formed by utilizing gelators (such as particles, fibers, or polymers) to generate a 3D network and encapsulate organic molecules [16, 17, 18, 19]. By adjusting the networks' structures, the morphology and physicochemical properties of organogels can be modified, and the encapsulation capabilities and release rate of organic molecules can be regulated [20, 21, 22]. Organogels provide significant advantages in the delivery of active ingredients [23, 24, 25], including high customizability. simple preparation [26], ease of modification [27, 28], and high biocompatibility [29]. These attributes have led to their widespread application in food and medical sectors [30, 31]. Compared with rigid carriers such as silica nanoparticles or polymer microcapsules with fixed geometries, this morphological regulation significantly influences the spreading behavior of the carriers across leaf microstructures, endowing the carrier with excellent interface compatibility. The resulting interfacial adaptability facilitates a superior foliar affinity, thereby enhancing the retention and efficacy of the active ingredients. However, the organogels are rarely utilized and reported in the pesticide field, since pesticide delivery faces complex and varied scenarios and requires unique performance differing from other pharmaceuticals [32], such as the uniformity of leaf surface coverage, adaptability to natural adversities like rainfall, and the negative impacts on non‐target organisms and the environment [33, 34]. These requirements present significant challenges in designing capable organogel materials for pesticide delivery.

**FIGURE 1:**
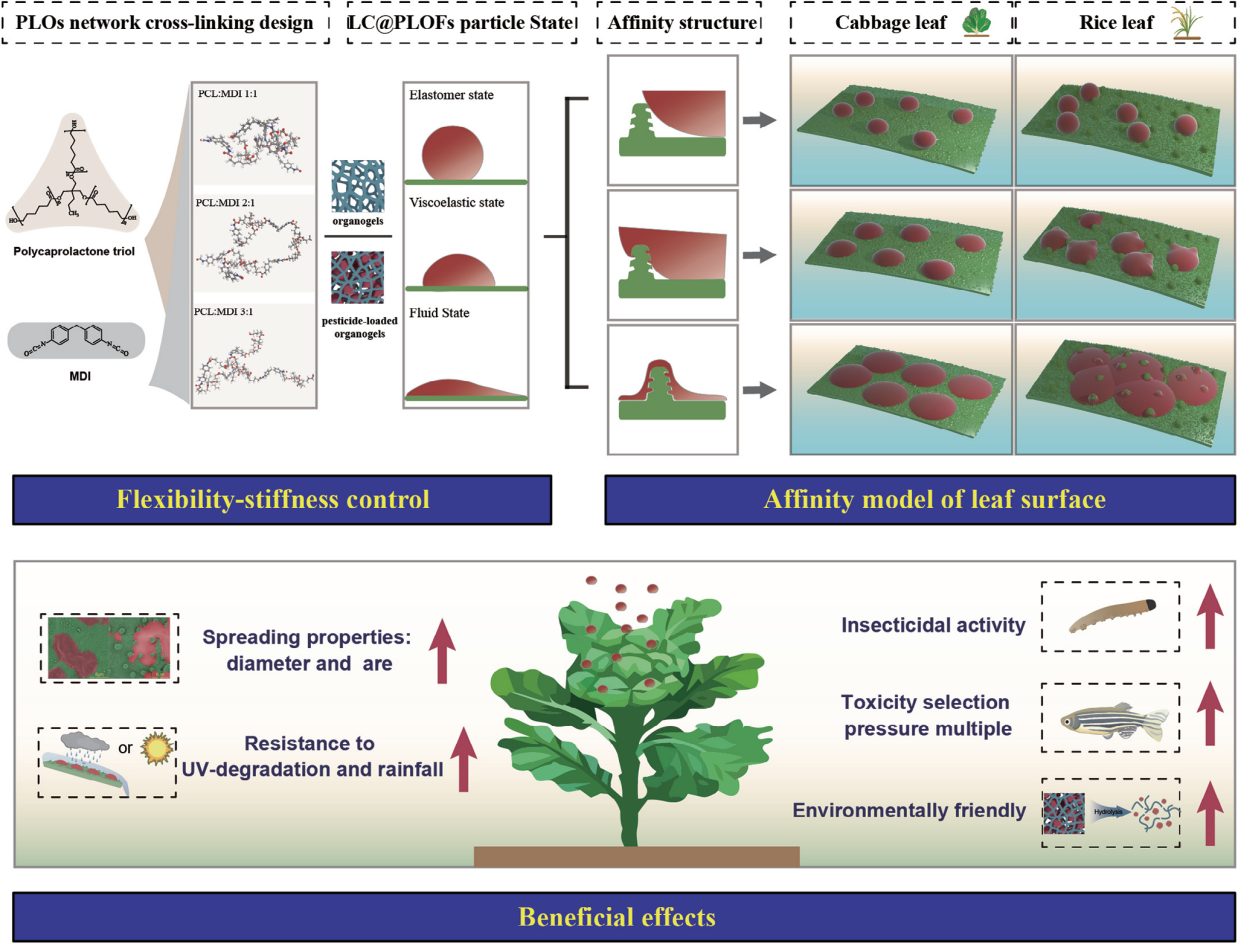
Design principle of PLOs and beneficial effect of PLOFs.

In this article, we developed a general technique lean on an innovative pesticide‐loading mechanism toward pesticide‐loaded organogels (PLOs) with high loading efficiency. By adjusting the reaction ratio of xylene methane diisocyanate (MDI) to polycaprolactone (PCL), the mesh sizes and mechanical properties of polyurethane networks can be tailored, presenting substantial potential for improving the precision of pesticide delivery. Through extensive molecular dynamics (MD) simulations and experiments, the impact of varying reaction ratios on the structure of the crosslinked network and mechanical performance of PLOs were determined, and the underlying principle of PLOs' efficient pesticide loading was revealed. Moreover, we established a water‐based formulation technology platform, namely the pesticide‐loaded organogels formulations (PLOFs), using PLOs materials to construct a pesticide delivery system. The micron‐scale PLOFs synthesis units were constructed in an aqueous phase by homogeneous dispersion, and the instant synthesis of PLOFs micron particles and synchronous pesticide loading were realized. We systematically evaluated the pesticide delivery performance of PLOFs in foliar environments, its potential for pest and disease control, and the risks to non‐target organisms, thereby opening new research avenues for the application of organogel materials in the agricultural field (Figure 1).

## 2 Results and Discussion

### 2.1 Design Principle and Performance Characteristic of PLOs

To obtain an efficient delivery‐type carrier, the strong loading and flexibility adjustment of gel‐type carriers need to be solved. Rational network design leveraging molecular engineering is promising to overcome these drawbacks, since the structure of porous polymer network is decisive for the performances of network and steric hindrance effect between network and pesticide [35, 36]. In designing the polymer network, polyurethane is commonly employed because of their robust mechanical properties and strong affinity, serving as the pesticide‐carrier [37]. However, the reaction of crosslinking strength is adversely influenced by soft segment structure [38]. For instance, the linear network formed by the reaction of diols is often not conducive to the design of pesticide‐carrier networks due to issues related to adhesion and the stability of pesticides controlled‐release. Thus, we ingeniously employed the PCL with three hydroxyl groups, to prepare and tailor highly‐crosslinked networks because it possesses high functionality and reactivity.

**FIGURE 2:**
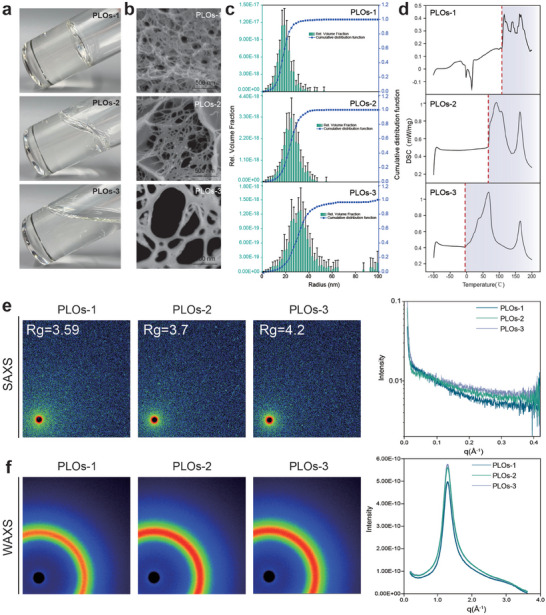
Comparison of the performance characteristics of three PLO samples. (a) Macroscopic states of PLOs samples. (b) Cryo‐scanning images of PLO. (c) The pore size analysis of PLOs was simulated and fitted according to SAXS data. (d) DSC analysis of the thermal stability of PLOs. (e) SAXS scattering image and characteristic curves of PLOs. (f) WAXS scattering image and characteristic curves of PLOs.

As shown in Figure 2a, Table S1 and VideoS1, as the ratio of PCL and MDI monomers changed from 1:1 to 2:1 and then to 3:1, the macroscopic state of the polymer evolves from a solid state (PLOs‐1) to a viscoelastic state (PLOs‐2), and eventually to a fluid state (PLOs‐3). The reason for the change in mechanical properties might be the change in the network structure caused by the variation in the monomer ratio. In PLOs‐1, there is more MDI and more rigid groups, thus it shows a solid state; while in PLOs‐2 and PLOs‐3, as the proportion of MDI decreases, the number of flexible groups increases, so they tend to change to a fluid state. The successful formation of the carbamate structure confirms the polymerization process, and the predominantly aliphatic long‐chain molecular structure ensures the material retains hydrophobic properties (Figure S1 and VideoS2).

Furthermore, by cryo‐scanning to observe the microscopic structure, we found that all three organogels exhibited a network‐like distribution(Figure 2b). The dense degree of the organogel network: PLOs‐1 > PLOs‐2 > PLOs‐3. The pore size distribution was simulated and fitted based on the scattering data, as shown in Figure 2c. The trend of the pore size was consistent with that of the cryo‐scanning images. The differential scanning calorimetry (DSC) results in Figure 2d further validate these findings, with PLOs‐1 exhibiting distinct thermal transition characteristics and a higher melting point, while PLOs‐3′s lower thermal transition temperature aligns with its fluid properties.

Wide‐angle X‐ray scattering (WAXS) and Small‐angle X‐ray scattering (SAXS) techniques are extensively used to analyze the microstructures of soft materials, such as polymers and gels [39, 40, 41]. We utilized SAXS to analyze the microstructures of the three PLOs samples (Figure 2e). The radius of gyration (Rg) characterizes the relationship between scattering intensity and the scattering vector q, providing an overview of particle dimensions. The Rg values for PLOs‐1, PLOs‐2, and PLOs‐3 are 3.59, 3.7, and 4.2, respectively. Rg is determined by the aggregation of the organogel network, where increased PCL content leads to greater crosslinking flexibility and an increase in Rg. Concurrently, the WAXS curves of the PLOs samples exhibited scattering peaks at 4.83 Å, likely corresponding to the benzene ring structure or carbamate group (Figure 2f) [19]. Additionally, X‐ray diffraction (XRD) analysis indicates that all PLOs samples exhibit an amorphous structure (Figure S2).

In conclusion, the three samples demonstrated significant differences in their structural, thermal, mechanical, and rheological properties. These findings lay the groundwork for further optimization of polyurethane materials and the expansion of their applications, such as in pesticide carrier materials.

### 2.2 Size Matching Between the Mesh of PLOs Network and LC

The changes in network structure caused by variations in monomer ratios lead to differences in macroscopic mechanical properties, which are difficult to characterize experimentally at the microscopic level. Molecular dynamics (MD) simulations can directly reveal the microstructure, providing an important basis for understanding the rigid‐flexible states of organogels and the pesticide‐loading mechanism.

**FIGURE 3:**
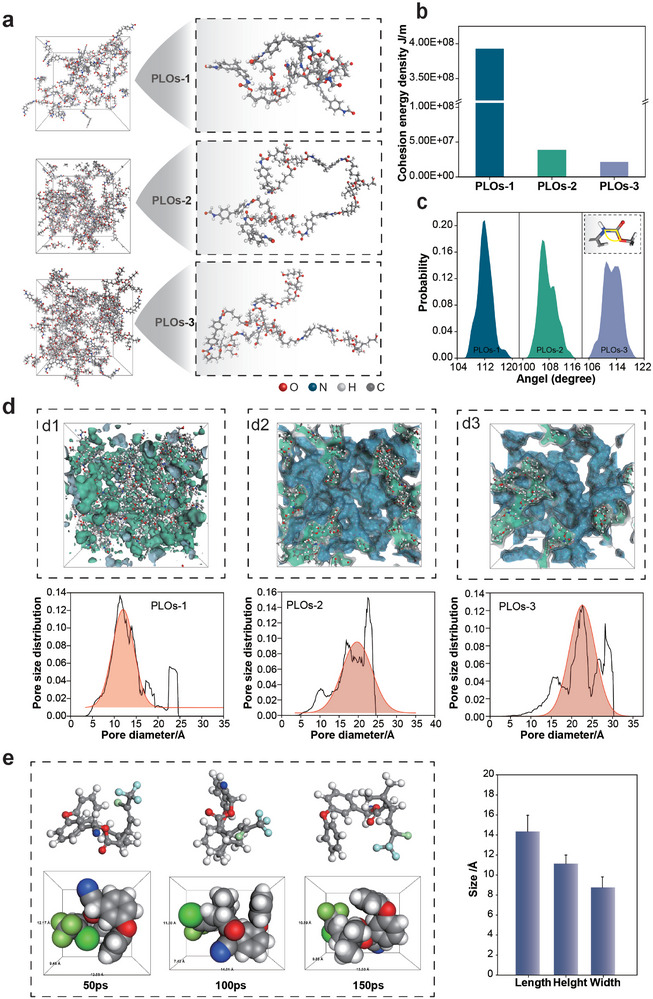
Network cross‐linking design and pesticide‐loading mechanism of PLO. (a) MD simulation of the PLOs network cross‐linking. (b,c) Cohesion energy density and Angle distribution diagram of carbamate bonds of PLOs. (d) Pore‐size distributions (PSD) of PLOs derived from pore statistics of the MD‐constructed networks (black curves). The orange shaded regions show Gaussian fits to the dominant peak region of the PSD, used to extract representative characteristic pore‐size ranges for comparison. (e) MD simulation of pesticide molecules and size characteristics.

The three cross‐linked networks are shown in Figure 3a. The intermolecular forces in these networks can better explain the formation of the flexible‐rigid states. Initially, as illustrated in Figure 3b, PLOs‐1 exhibits the highest cohesion energy density, indicating the strongest intermolecular interactions, directly correlating with its enhanced solid‐state characteristics. In contrast, PLOs‐2 and PLOs‐3 demonstrate lower cohesion energy densities, with PLOs‐3 having the least, which provides a kinetic foundation for its fluid state. This observation aligns with the solubility parameters: higher cohesion energy densities correspond to stronger intermolecular forces and tighter segmental bonding, leading to reduced solubility (Figure S3). Figure S4 shows that intermolecular electrostatic and van der Waals interactions are most pronounced in PLOs‐1. In the analysis of urethane bond angle distributions (Figure 3c), PLOs‐1 reveals a more concentrated and ordered bond angle distribution, reflecting robust structural integrity.

Furthermore, the simulation tensile test also confirmed the above viewpoint. Figure S5 shows the structural evolution of the material during stretching. Under the same stress, as the intermolecular interactions increase, PLOs samples exhibit smaller strain levels. The distinct stress–strain responses of different PLOs samples can be attributed to variations in polymer chain configurations. The rotational stress‐strain curves also exhibit the same pattern (Figure S6).

Actually, the interaction and compatibility between organogels and pesticides are not only decided by the flexible‐rigid chains, but also related to the structures of the entire network architecture. To further compare the compatibility between organogels and pesticides, the models of entire network trapping pesticide molecules were established. We simulated the microstructure of three PLOs cross‐linked networks, and then made statistics on the pore distribution. As shown in Figure 3d, in our design, three types of networks with different flexible‐rigid states were formed. The results indicate that the pore size distribution tends toward a normal distribution. We conducted data fitting based on the statistical results and identified the main pore sizes. The pore sizes of the three organogel samples were mainly distributed within the range of 5–20 Å (PLOs‐1), 10–30 Å (PLOs‐2), and 15–30 Å (PLOs‐3).

The poor stability of organogels is considerably resulted by the severe migration of pesticides owing to size mismatch between the network and pesticides molecules [2, 16, 17]. To obtain stable pesticide‐loading materials, the steric hindrance effect is vital, which requires precise size matching between pesticide molecules and meshes of organogels network [35, 36]. Therefore, the molecular conformation of pesticide molecules was analyzed by MD simulation for 150 ps (Figure 3e; Figure S7). The 3D size of Lambda‐cyhalothrin (LC) and Pyr molecules was calculated based on their atomic radius and spatial coordinates. The length, width, and height of the LC molecule are 16.5, 7.9, and 11.2 Å, respectively. To achieve the steric hindrance effect, the size of pesticides molecule should be less than the mesh size of organogels network. These findings suggest that in PLOs‐3, the largest pore size allows unrestricted multidirectional migration of LC molecules since their sizes are totally smaller than the largest pore size; in PLOs‐2, the medium‐sized aperture confines LC molecules to a specific angular range; in LC@PLOs‐1, the smallest aperture further restricts diffusion to an even narrower angle, which endows PLOs‐1 gels with good stability. This demonstrates that the compatibility degree between the network and pesticides decreases in the order of PLOs‐1>PLOs‐2>PLOs‐3. Nevertheless, the actual capturing efficacy of PLOs toward pesticides requires further research via subsequent experiments.

### 2.3 Performance Characteristics of LC@PLOs After Loading Pesticides

**FIGURE 4:**
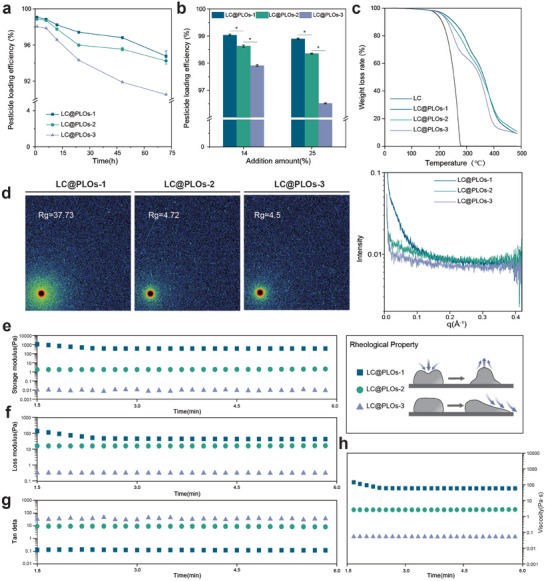
Comparison of the performance characteristics of LC@PLOs after loading pesticides. (a) Pesticide loading efficiency of LC@PLOs at different times (b) Pesticide loading efficiency of LC@PLOs at different addition amounts (c) TGA images of LC@PLOFs samples. (d) SAXS scattering image and characteristic curves of PLO samples. (e–g) Rheological properties and (h) viscosity analysis of PLOs samples.

Before comparing the encapsulation effect of the organogel network on pesticides, it is necessary to clarify that the pesticide loading capacity and loading time of the organogel network are the key factors affecting the actual usage effect. The pesticide loading capacity is related to the formulation content, and the loading time is related to the sustained‐release and controlled‐release effect. Therefore, the study systematically compared the pesticide loading efficiency of three organogel networks under different loading times (Figure 4a) and different pesticides loading capacities (Figure 4b). As shown in Figure 4a, the results indicate that as time progresses, the rigid structure of LC@PLOs‐1 has a better binding effect on pesticides. At 72 h, its loading efficiency can still maintain 94.7%. Overall, the three organogel networks with different rigidity (72 h) all achieved a binding efficiency of over 90% for the pesticide. As shown in Figure 4b, the results indicate that the binding strength of the three organogels to the pesticide, from strong to weak, is: LC@PLO‐s1 > LC@PLOs‐2 > LC@PLOs‐3. When the addition amount is 25%, the pesticide binding efficiencies of LC@PLOs‐1, LC@PLOs‐2, and LC@PLOs‐3d are 98.9%, 98.4%, and 96.5%, respectively. The three different networks still maintain a high binding state at a relatively high addition amount. Therefore, the results show that organogels' effectiveness in capturing and binding pesticide molecules is closely linked to size differences and structural compatibility between the two molecule types. Furthermore, all three carriers exhibit thermal protection properties against pesticides (Figure 4c).

Further, through microscopic molecular structure analysis, the changes in the gel network before and after pesticide‐loading were investigated. The Rg data for six samples of PLOs and LC@PLOs are shown in Figure 4d and Figure S8. Before the addition of pesticide molecules, the Rg is determined by the aggregation of the crosslinking network; as the PCL content increases, the cross‐linked chains become more flexible, leading to an increase in Rg. After adding pesticide, the Rg values of LC@PLOs‐1, LC@PLOs‐2, and LC@PLOs‐3 increased to 37.73, 4.72, and 4.5, respectively, to varying degrees. The Rg of LC@PLOs‐1 increased significantly by 10 times. This is attributed to the efficient binding of the pesticide molecules, which helps maintain a high‐strength stability. Furthermore, by comparing the scattering curves before and after the application of the pesticides, it was found that there were no absorption peaks, indicating that there were no aggregated or micro‐phase separation structures. This is further supported by the macro state observations of PLOs and LC@PLOs (Figure S9).

Figure 4e–g presents the variations in storage modulus (E′), loss modulus (E′′), and loss coefficient (tan δ) as a function of time for the three samples. Rheological experiments indicate that the loss factor of PLOs‐1 is lower than that of PLOs‐2 and PLOs‐3, suggesting that PLOs‐1 primarily exhibits elastic behavior under stress, effectively storing energy. As the degree of crosslinking decreases, the loss factors of PLOs‐2 and PLOs‐3 increase, showing stronger viscous behavior. Complex viscosity, an important parameter reflecting the flow characteristics of materials, is highest for PLOs‐1, confirming its structural stability, while the lowest viscosities of PLOs‐2 and PLOs‐3 highlight their excellent fluidity and processability (Figure 4h).

The intelligent design of pesticide carriers, through precise modulation of their mechanical properties, more effectively influences the performance of active ingredients at the microscopic level. The “one‐step” loading mechanism streamlines the traditional pesticide loading process, significantly enhancing loading efficiency. Compared to conventional adsorption and encapsulation methods, this innovative strategy demonstrates exceptional advantages in improving drug delivery efficiency and precision.

### 2.4 Preparation Process of LC@PLOFs

**FIGURE 5:**
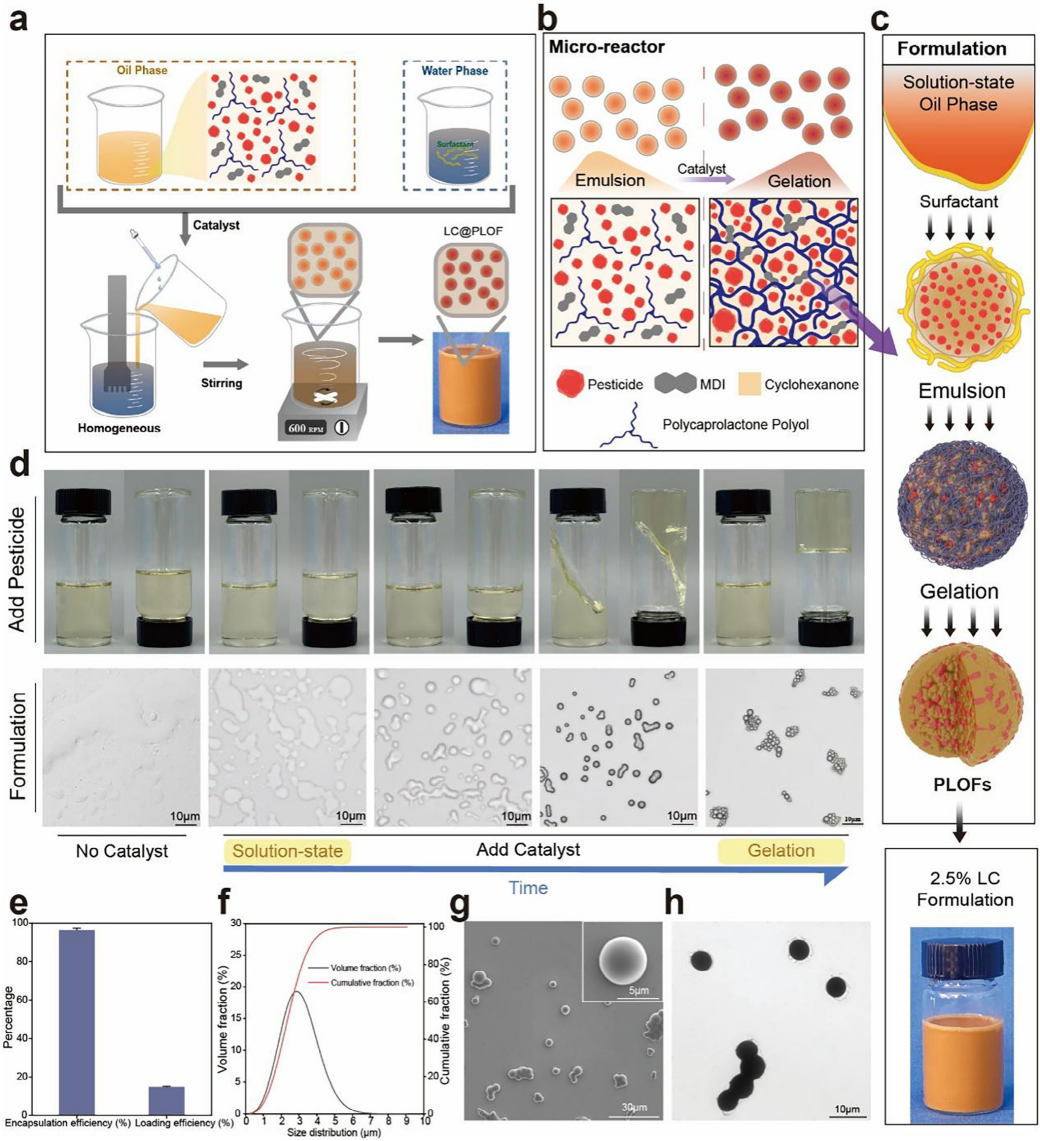
Preparation and characterization of LC@PLOFs. (a) Schematic diagram of the water‐based preparation of LC@PLOs formulation. (b) Schematic diagram of the changes in the internal structure of the micrometer‐sized reaction space from droplets to microspheres before and after the addition of a catalyst. (c) Schematic diagram of the formation of PLOFs particles through homogenization emulsification technology under the action of surfactants. (d) Corresponding change process of the macroscopic oil phase and microscopic particles after the addition of the catalyst. (e) Encapsulation efficiency and loading efficiency of LC@PLOFs. (f) Size distribution of LC@PLOFs (g) SEM and (h) TEM image of LC@PLOFs.

Figure 5a–c illustrates the preparation of PLOs microparticulate through the construction of microreactor units based on the principle of solution polymerization. LC was used as the model pesticide. First, the reaction monomers in the organic phase are driven to react by a catalyst. Subsequently, the organic phase, while maintaining a weak mechanical state, is uniformly mixed with the aqueous phase and homogenized. Multiple types of surfactants are utilized to form stable, micron‐sized reaction units with a dispersed state in the aqueous phase. Non‐ionic surfactants can form a dense protective layer between the organic and aqueous phases, maintaining the stability of the internal reaction space of the organic phase. At the same time, ionic surfactants inhibit collisions and aggregation between the micron‐sized reaction units through charge repulsion and steric hindrance, thereby ensuring the smooth cross‐linking reaction of the active monomers within the reaction units. Ultimately, gelation occurs within the micron‐sized reaction units, and the pesticide is loaded efficiently, resulting in a uniform and stable LC@PLOFs formulation. The state changes during the organic phase reaction prove that the pesticide and solvent are simultaneously adsorbed and loaded when gelation is completed (Figure 5d). This simple and rapid process significantly reduces the difficulty of industrial application of pesticide carrier technology.

The preparation method was proven to meet the application requirements for pesticide‐loaded formulations by the basic characterization of the formulation (Figure 5e–h). The encapsulation efficiency of the LC@PLOFs was 96.4%, and the loading efficiency reached 14.8%, indicating excellent pesticide‐loading capacity. The particle size exhibited a unimodal distribution, with an average diameter (D_50_) of 2.34 µm. SEM images revealed that the LC@PLOFs possessed a smooth, uniform surface with good dispersion. TEM analysis showed that the internal structure of the LC@PLOFs was homogeneous and solidified, suggesting that the reactions in the organic phase proceeded efficiently. Consequently, a uniform and stable pesticide‐loaded material was successfully obtained.

### 2.5 Basic Properties of LC@PLOFs

**FIGURE 6:**
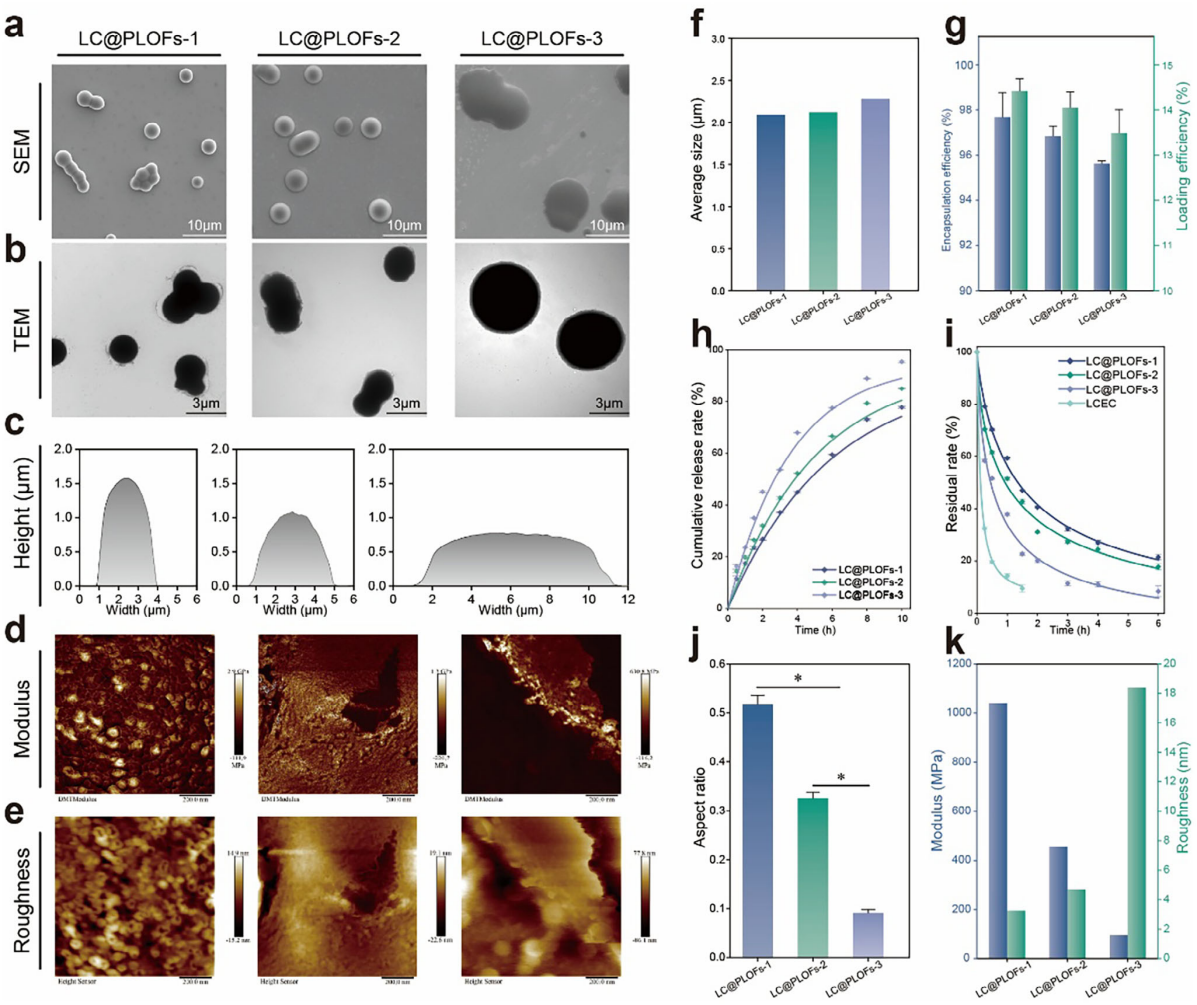
Characterization of basic properties of LC@PLOFs. (a) SEM, (b) TEM, and (c) AFM images of morphological characterizations of LC@PLOFs. (d) Modulus and (e) roughness images of mechanical properties of LC@PLOFs. (f) Average particle sizes, (g) encapsulation efficiency and pesticide‐loading amount, (h) release behavior, (i) UV resistance simulation experiment, (j) aspect ratio, (k) moduli and roughness of LC@PLOFs samples. All experiments were performed three times, and error bars represent standard deviation (SD). ^*^, P *p* < 0.05.

Therefore, three LC@PLOFs suspension concentrates (SC) were prepared using LC@PLOs with different flexible states. The particle sizes of the LC@PLOFs particles are uniformly around 2 µm, exhibiting a unimodal particle size distribution (Figure S10). The mean particle sizes (D_50_) for LC@PLOFs‐1, LC@PLOFs‐2, and LC@PLOFs‐3 were 2.09, 2.12, and 2.28 µm, respectively (Figure 6f). All three LC@PLOFs formulations exhibited encapsulation efficiencies exceeding 95%, demonstrating a high pesticide encapsulation capacity (Figure 6g).

The morphological changes and performance differences of LC@PLOFs were further analyzed. The morphological changes of different flexibres LC@PLOFs before and after water evaporation are shown in Figure S11. All three LC@PLOFs samples exhibit a micron spherical state in water and have good dispersion in water. LC@PLOFs can maintain their spherical shape in water due to the interfacial tension at the solid‐liquid interface; however, this interfacial force dissipates upon the evaporation of water. Under the combined influence of gravity and adhesion, different LC@PLOFs samples undergo distinct morphological changes. SEM and TEM images show that all LC@PLOFs have smooth and continuous surfaces and uniform interiors (Figure 6a,b). The results of 3D micromorphology parameters of LC@PLOFs using AFM are presented in Figure 6c. With the increase in aspect ratio, the morphology of LC@PLOFs gradually changes from spherical to flattened state (Figure 6j). The LC@PLOFs‐3 prepared from the fluid state LC@PLO show a flatter interface spreading state, and the spreading diameter and spreading of LC@PLOFs‐3 on the interface are increased by approximately 1.86 and 7.9 times higher than that of LC@PLOFs‐1(Figure S12). The deformability and spreading properties of these particles are advantageous in enhancing the initial exposure level and the rapid efficacy of the pesticide loading system, which is of significant importance for improving the utilization efficiency of pesticides. When combined with the modulus and roughness of LC@PLOFs‐3, it exhibits a higher degree of flexibility and a lower surface roughness (Figure 6d,e,k), indicating its promising potential for foliar affinity.

Additionally, three LC@PLOFs samples exhibited pronounced controlled‐release behavior for pesticides (Figure 6h). There are discernible differences in the release kinetics of LC@PLOFs, with the release rate following the order LC@FC‐3 > LC@FC‐2 > LC@FC‐1. These findings indicate that alterations in the molecular structure of LC@PLOFs not only modulate their mechanical properties but also enable precision of the pesticide release behavior, which is of great significance for the precise delivery of pesticides.

Furthermore, a simulation experiment was conducted to assess the UV resistance of three LC@PLOFs formulations (Figure 6i). Following 1.5 h of UV irradiation, the residual pesticide concentrations in the LC@PLOFs were found to be 2.4 to 5 times greater than those in the EC. The elastomeric LC@PLOFs‐1 and the viscoelastic LC@PLOFs‐2 exhibited superior UV resistance when compared to the fluid LC@PLOFs‐3, suggesting that a thicker carrier matrix can mitigate UV penetration and thereby decrease the degradation efficiency of pesticides within the LC@PLOFs. The advantageous UV resistance of the three LC@PLOFs formulations also enhances the extended efficacy period of the pesticides in complex field environments.

### 2.6 Foliar Affinity of LC@PLOFs

**FIGURE 7:**
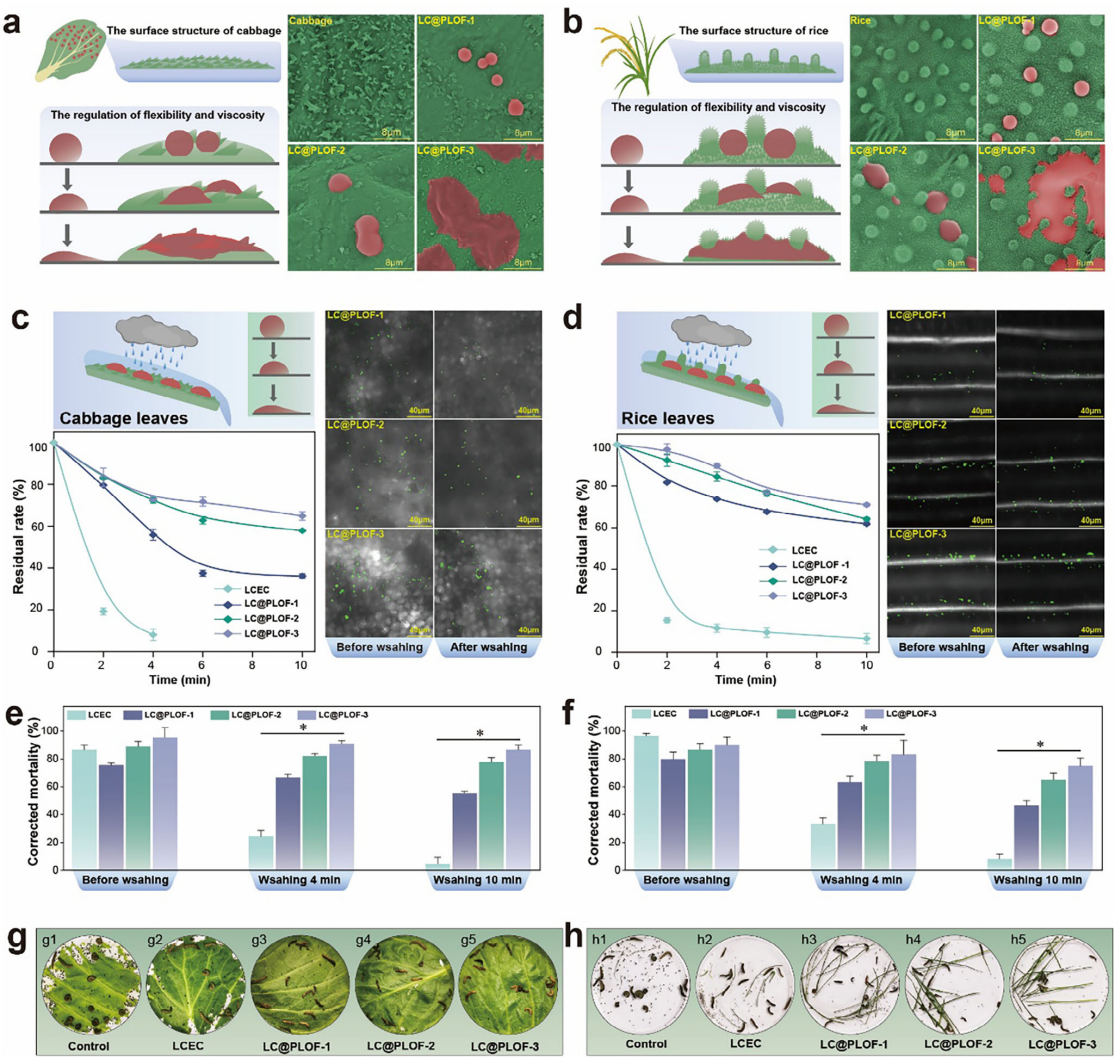
Affinity of LC@PLOFs on (a) cabbage leaves and (b) rice leaves. Pesticide residual rate and distribution behavior of LC@PLOFs before and after washing on (c) cabbage leaves and (d) rice leaves. (e,f) Insecticidal activity and (g,h) leaf state of LC@PLOFs before and after washing on cabbage leaves and rice leaves. All experiments were performed three times, and error bars represent standard deviation (SD). ^*^, P *p*< 0.05.

The flexible morphological transformation capability of LC@PLOFs enables the pesticide‐loading system to achieve enhanced adaptability to the micromechanical structure of the leaf surface, thereby improving leaf surface affinity and biological activity [42, 43]. To ascertain the effect and universality of LC@PLOFs' flexibility on foliar affinity, cabbage and rice leaves, which exhibit distinct microstructures, were employed as model systems. Scanning electron microscopy (SEM) observations (Figure 7a,b) reveal that as LC@PLOFs transition from a solid to a fluid state, they deform and spread over a larger area of the leaf surface, forming a flat, spreading structure. This facilitates the formation of a larger contact area and a more intimate mosaic state with the leaf surface's microstructure. The leaf surface of rice is covered with complex micrometer‐sized protrusions. Observations using a SEM revealed that these intricate microscopic structures provided additional affinity sites during the LC@PLOFs particles' spreading process. Based on this, it can be speculated that this more compact interface adaptation state helps the LC@PLOFs particles establish a more stable adhesion on the leaf surface, thereby significantly enhancing the retention ability of the carrier under climatic adversity.

The residual rates of pesticides and their distribution behaviors before and after washing, as depicted in Figure 7c,d, reveal that the pesticide residual rates of the three LC@PLOFs samples were significantly greater than those of LCEC over the same washing duration. The fluid state LC@PLOFs‐3 exhibited superior retention on the foliar surface and the highest resistance to washout. Notably, the retention rates of pesticides on rice leaves were significantly higher than those on cabbage leaves at equivalent washing times. For LC@PLOFs‐1, after 10 min of washing, the pesticide residual rate on rice leaves was approximately 25.7% greater than that on cabbage leaves. This is attributed to the more complex microstructure of the rice leaf surface, characterized by features such as trichome structure, which not only act as barriers for LC@PLOFs but also provide additional affinity structures and sites during the spreading process, thereby establishing a more robust resistance to rain erosion on the leaf surface. Furthermore, the polymer coating that forms on the leaf surface after the spreading of LC@PLOFs may also contribute to its resistance against rain wash‐off (Figure S13). As water evaporates from the leaf surface, the LC@PLOFs in the liquid phase can provide a protective coating for the applied site. By enhancing the hydrophobicity of the leaf surface at these locations, the likelihood of rain contact is reduced, thereby minimizing the potential for pesticide loss.

As anticipated, the exceptional foliar affinity of LC@PLOFs significantly enhances their insecticidal efficacy under rain conditions (Figure 7e). Prior to washing, there was no significant difference in insecticidal efficacy between LCEC and the LC@PLOFs formulations. The LC@PLOFs‐3 sample, with its superior deformation and spreading capabilities, demonstrated excellent rapid insecticidal effectiveness, as it significantly improved the contact efficiency between the pesticide and the target insect. Following a 10‐min wash, the mortality rate of insects treated with LCEC decreased to 4.5% and essentially lost its insecticidal activity. In contrast, the mortality rates of LC@PLOFs‐1, LC@PLOFs‐2, and LC@PLOFs‐3 were 55.5%, 82.2%, and 86.7%, respectively. Figure 7g depicts the state of the test insects and the protective effect on cabbage leaves after a 4‐min wash. Moreover, considering the complexity and diversity of leaf surface structures, washing simulation experiments were also conducted on rice leaves, which possess a more intricate microstructure (Figure 7f). Overall, the trend in the insecticidal efficacy of LC@PLOFs following continuous washing is fundamentally consistent with their wash resistance properties.

### 2.7 Biological Activity of LC@PLOFs Under Adverse Conditions

**FIGURE 8:**
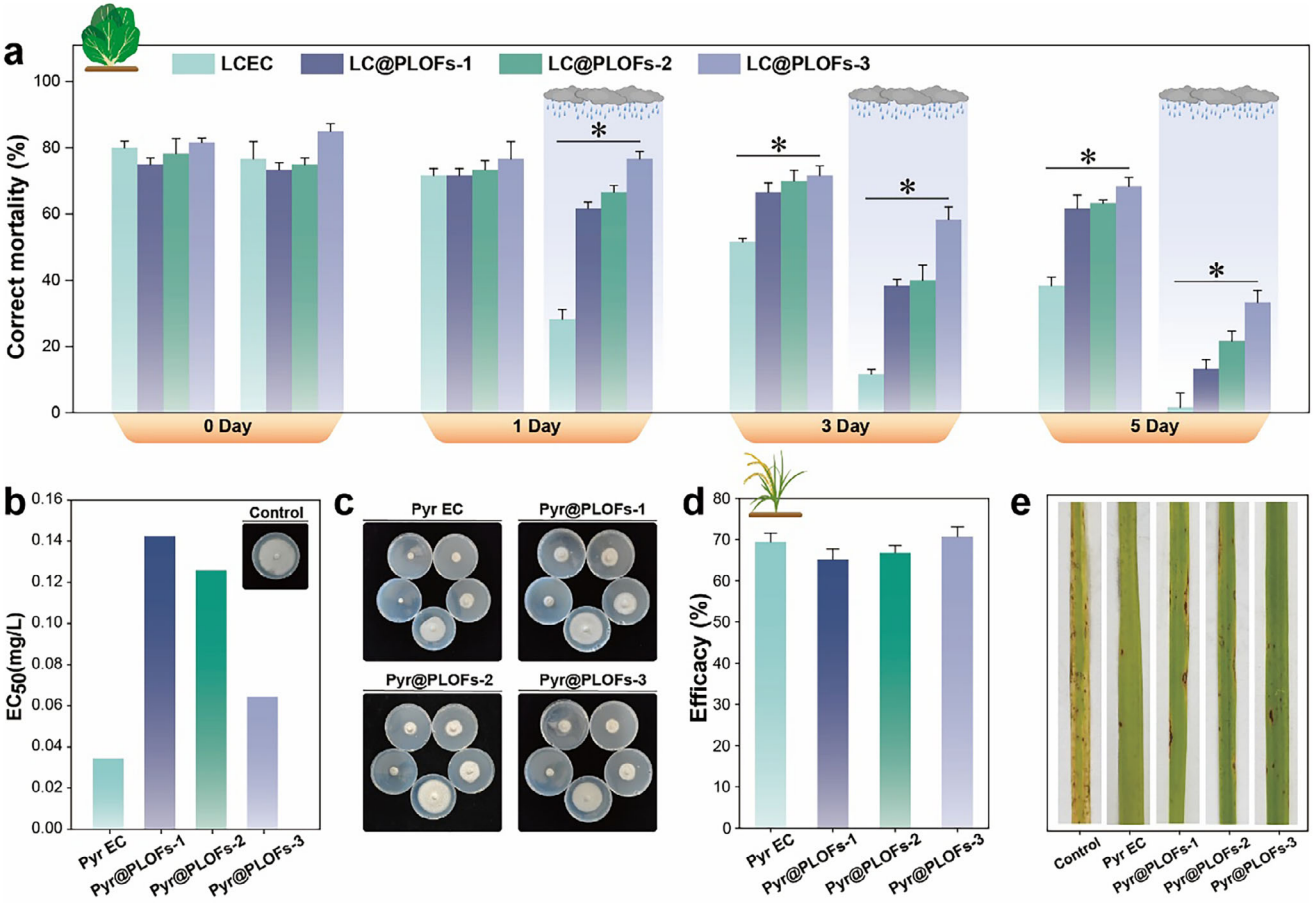
Efficiency of LC@PLOFs in the greenhouse. (a) Insecticidal activity of LC@PLOFs after washing in the greenhouse. (b) Magnaporthe oryzae EC_50_ values, (c) bacteriostatic state, (d) efficacy, (e) leaf state of rice. All experiments were performed three times, and error bars represent standard deviation (SD). ^*^, P*p* < 0.05.

Figure 8a presents the comparative insecticidal efficacy of LC@PLOFs and LCEC in greenhouse settings. In unwashed conditions, the mortality rate of the LCEC treatment decreased to 38.3% by the 5th day, whereas the LC@PLOFs treatment maintained a mortality rate exceeding 60%. This indicates that under unwashed conditions, the decline in insecticidal efficacy is predominantly attributed to the UV‐mediated degradation of pesticide components within the greenhouse environment. The pronounced large‐area deformation and spreading capabilities of LC@PLOFs‐3 mitigate the rapid decrement in insecticidal efficacy resulting from UV‐induced pesticide degradation to a significant degree. Conversely, the mortality rate of the LC@PLOFs treatment was approximately 2–3 times higher than that of the LCEC treatment on the first day post‐application, under washed conditions. This observation further confirms that washing accelerates the loss of pesticide dosage on the leaf surface, leading to a substantial reduction in the insecticidal efficacy of LC EC. Additionally, as the frequency and duration of washing increase, the overall insecticidal efficacy of the LC@PLOFs treatment is notably diminished compared to that observed without washing. This underscores the more pronounced impact of washing factors on leaf pesticide components compared to UV factors, highlighting the necessity for attention and improvement in the management of leaf pesticide residues.

To ascertain the superiority and universality of PLOF in diverse plant protection contexts, we synthesized a 9% Pyr@PLOFs formulations for the assessment of its indoor fungicidal activity and greenhouse efficacy against rice blast. The EC_50_ values for Pyr EC, Pyr@PLOFs‐1, Pyr@PLOFs‐2, and Pyr@PLOFs‐3 were determined to be 0.0345 mg/L, 0.1425 mg/L, 0.1259 mg/L, and 0.0644 mg/L, respectively (Figure 8b). The elevated fungicidal activity of Pyr EC can be attributed to the direct contact of the pesticide active ingredients with the PDA plate. Conversely, the fungicidal efficacy was enhanced with the increased flexibility of the carrier material, and the fungicidal activity of Pyr@PLOFs‐3 was particularly pronounced. The enhanced initial exposure efficiency contributed to the increased fungicidal activity of Pyr@PLOFs‐3 (Figure 8c). Furthermore, the fungicidal activity of Pyr@PLOFs against rice blast in a greenhouse setting was investigated. In comparison to the control, all treatments exhibited a significant reduction in the severity of disease spots (Figure 8d). Notably, the efficacy of Pyr@PLOFs‐3 was marginally superior to that of the Pyr EC.

The complex fluctuations in field environmental parameters typically have a profound impact on the efficacy of pesticides against pests and diseases [44, 45]. we evaluate the field efficacy of the LC@PLOFs and Pyr@PLOFs systems (Figures S14 and S15). The comprehensive control efficacy of the flexible pesticide loading system was found to be superior to that of the conventional EC formulations, thereby significantly enhancing the field efficacy of the treatment.

### 2.8 Non‐target Safety of LC@PLOFs

**FIGURE 9:**
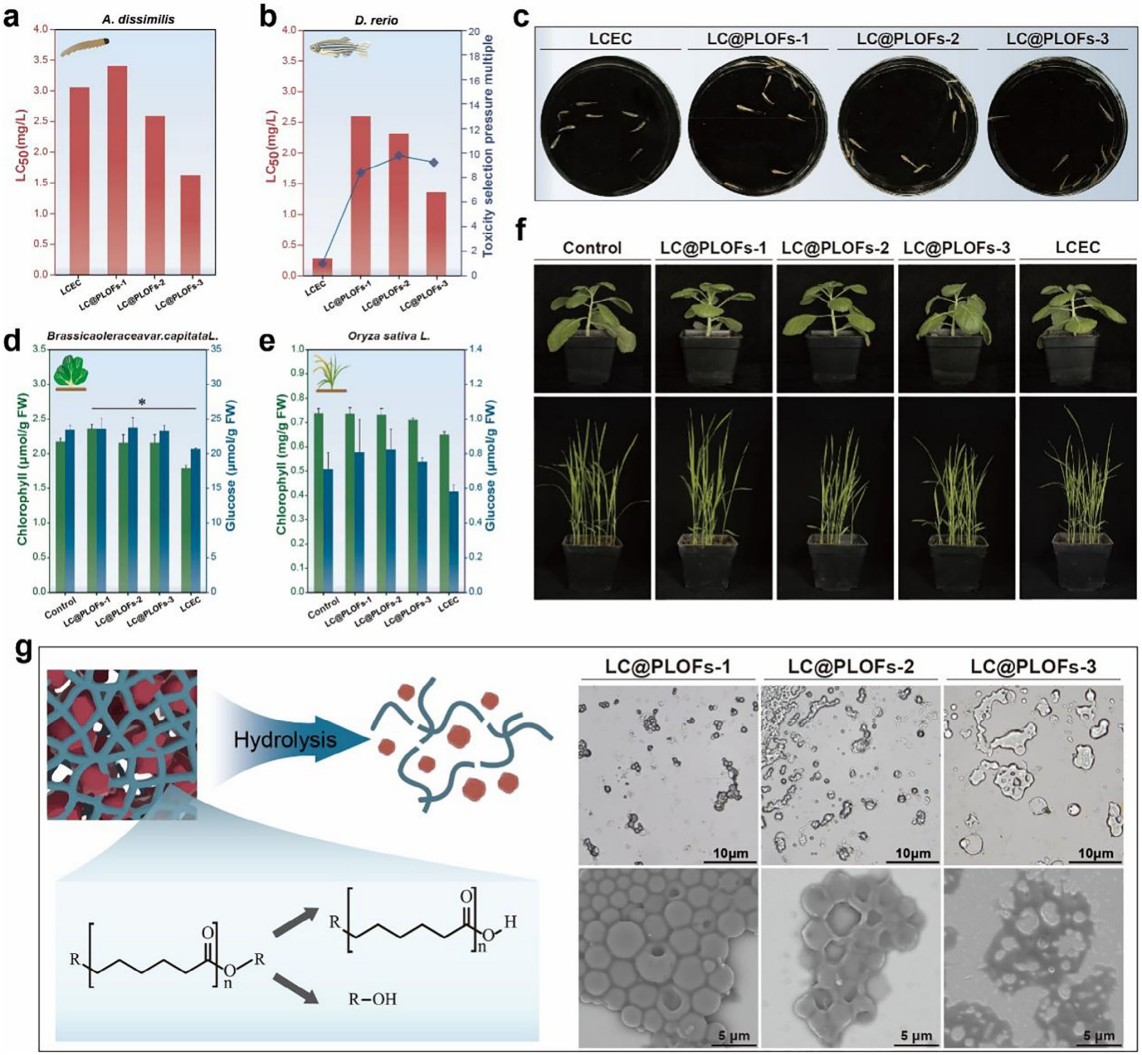
Non‐target safety of LC@PLOFs. (a) LC_50_ values of test worm, (b) LC_50_ value, toxicity selection pressure multiple, and (c) survival status of zebrafish according to Pyr@PLOFs in the greenhouse. Chlorophyll and glucose content in (d) cabbage and (e) rice, (f) growth status of cabbage and rice. (g) Hydrolytic state of LC@PLOFs. All experiments were performed three times, and error bars represent standard deviation (SD). ^*^, P*p* < 0.05.

Enhancing the selectivity of the pesticide loading system for targeted delivery is crucial for balancing the efficiency and safety of pesticides [46]. In this study, the paddy field plant protection scenario is used as a model to evaluate the toxic selectivity of LC@PLOFs based on their foliar insecticidal efficacy and aquatic toxicity (Figure 9a–c). The LC_50_ values for LCEC, LC@PLOFs‐1, LC@PLOFs‐2, and LC@PLOFs‐3 were determined to be 0.28, 2.6, 2.3, and 1.36 mg/L, respectively (Figure 9b). Figure 9c illustrates the zebrafish survival status under a uniform concentration, where LCEC caused significantly higher mortality. The LCEC resulted in a significant number of deaths. This observation aligns with the LCEC toxicity range reported by Huang et al. [47]. The disparity in toxicity is primarily attributed to the interfacial state of the active ingredients in water. While both formulations exist as micron‐sized spherical particles, the active ingredients in LCEC are fully exposed and exhibit high lipophilicity, leading to a potent affinity for the toxicological targets of zebrafish. In contrast, the LC@PLOF carriers maintain the pesticides in varying degrees of a bound state, effectively minimizing the exposure level and significantly reducing the risk to non‐target aquatic organisms. The toxicity selection pressure multiples for LC@PLOFs‐1, LC@PLOFs‐2, and LC@PLOFs‐3 were determined to be 8.40, 9.79, and 9.22, respectively (Figure 9b). The results indicate that the toxicity selection pressure of LC@PLOFs is higher than that of LCEC to varying extents, suggesting its potential to enhance efficacy while reducing toxicity.

The excellent toxic selectivity of the LC@PLOFs system is attributed to its ability to exhibit opposite pesticide exposure states in foliar and aquatic environments. On the leaf surface, it spreads out to enhance the pesticide exposure level, while in water, it contracts to reduce the pesticide toxicity level. This intelligent adjustment of the carrier morphology and pesticide exposure dose based on the characteristics of the environment improves the selectivity for target toxicity and represents an important technical approach for the precise delivery of pesticides.

Consequently, we investigated the impact of LC@PLOFs on crop photosynthetic pigments and their metabolites. As shown in Figure 9d–f, the chlorophyll and glucose contents in cabbage and rice were monitored following a 5‐day application of the pesticide. These findings indicate that the three LC@PLOFs samples, alongside their favorable leaf surface properties, exceptional insecticidal and fungicidal activities, also exhibit minimal adverse effects on plants, thus demonstrating their safety. Currently, the potential hazards of microplastics have garnered extensive international concern. The biodegradability of pesticide‐loaded materials is a critical issue that warrants ongoing consideration [48, 49]. The primary structural component of PLO is biodegradable polycaprolactone (PCL), which contains a large number of ester and ether bonds, conferring it with degradation potential [50, 51]. In this study, the hydrolysis state of LC@PLOFs was evaluated (Figure 9g). Notably, the LC@PLOFs‐3 formulations, which employs the highest content of PCL, are expected to be more readily degradable. Compared to conventional high molecular polymer materials, PLO offers the potential to mitigate environmental pollution risks and exhibits favorable environmental compatibility. While the integration of biodegradable PCL as a matrix effectively alleviates the long‐term ecological burden compared to persistent synthetic polymers, the inherent complexity of the MDI‐based cross‐linked network—and the potential release of degradation products and pesticide metabolites the necessity for continuous environmental monitoring. Future research focusing on the long‐term degradation kinetics and the synergistic toxicological profiles of these secondary products will be essential to fully elucidate the environmental fate of this organogel system and to ensure its safe, sustainable application in modern crop protection.

## 3 Conclusion

In summary, this study has developed an organogel based on the demand for precision pesticide delivery, capable of simultaneous rapid gelation and efficient pesticide loading in the organic phase. By constructing gelling units within micron‐scale reaction spaces, the micromization and formulation of pesticide‐loaded organogels are achieved. The mechanical properties of the organogel can be flexibly adjusted through its network molecular structure, thus precisely altering the topography of the pesticide‐loaded organogel to enhance structural adaptability and retention stability on biological interfaces, thereby improving pesticide utilization under adverse climatic conditions. Meanwhile, the flexible deformation characteristics of the carrier provide better delivery selectivity in complex crop protection scenarios, balancing target efficacy and non‐target safety. This technology shows promising applicability and versatility for agricultural chemicals, with low raw material costs and simple processes, offering significant potential for industrial scale‐up.

## 4 Experimental Section

### 4.1 Chemicals and Materials Information

Lambda‐cyhalothrin (LC) was provided by ShanDong Luba Chemical Co., Ltd. (96%, Shandong JiNan, China). Pyraclostrobin (Pyr) was provided by ShanDong Kangqiao Biotechnology Co., Ltd. (97%, Shandong Bin Zhou, China). MDI was purchased from Wanhua Chemical Group Co., Ltd. (99.6%, Shandong, China). Emulsifier #602 was purchased from Shandong Tiandao Biological Engineering Co., Ltd. (Shandong, China). PCL was provided by Juren Chemical Hitechnology Co., Ltd. (99.9%, Hunan, China). N‐butanol, N‐hexane, Methanol, Ethanol, Acetonitrile, dibutyltin dilaurate, cyclohexanonel (analytical grade), and fluorescein isothiocyanate (FITC) were purchased from Aladdin Industrial Corporation (America). Sodium lignosulfonate (SL, molecular weights: 1.0 × 10^4^−1.2 × 10^4^) was purchased from MeadWestvaco, Inc. (America).

### 4.2 Insect Source

The tested larvae were Agrotis ipsilon. Rearing condition: The insects were successively reared in the laboratory and fed artificial feed at 25 ± 1°C and 70 ± 5% relative humidity in 16:8 light/dark cycles. Third‐instar larvae were selected for bioassay and greenhouse experiments. Cabbage and rice were cultivated artificially in a greenhouse without exposure to any chemicals. The details of the feed formulation can be found in the Supporting Information.

### 4.3 Preparation of PLOs,, LC@PLOs and LC@PLOFs

According to the proportions in Table S1, the dosage of two reaction raw materials, PCL and MDI, was added to obtain PLOs with different flexible states.

According to the proportions in Table S2, First, 2.60 g of LC, 10.0 g of cyclohexanone, and a certain amount of PCL and MDI were weighed and mixed to generate an organic phase. The fully dissolved homogeneous organic phase was transferred to a sample bottle with a capacity of 25.00 mL, and 200 µl dibutyltin dilaurate was added to the sample bottle as the reaction catalyst. The changes of the appearance state of the reaction system were observed and photographed during the reaction process.

The LC@PLOFs preparation process is shown in Figure 4 and Table S3. Refer to the LC@PLOs preparation process to obtain the organic phase. The continuous phase was obtained by accurately weighing 2.00 g emulsifier 602# and 3.00 g sodium lignosulfonate, thoroughly mixed and dissolved in 50.00 g deionized water. Notably, to indicate the distribution of the sample particles on the organism, fluorescent samples were prepared using the same method but with the addition of 1 mL of 0.01% FITC cyclohexanone solution in the oil phase. Add the organic phase to the continuous phase, homogenizing the mixture at 8000 rpm for 1 min at 25°C. The emulsion was transferred to a glass bottle, this was done under magnetic stirring at 300 r/min for 2 h at room temperature. Deionized water to 100 g to obtain 2.6% LC@PLOFs.

According to the above process, the active ingredient of the pesticide was changed to add 9.28 g Pyr, and the dosage of PCL and MDI was added in accordance with the proportions in Table S3 to prepare 9% Pyr@PLOFs with different flexible states. Emulsifiable concentrate (EC) used in this research is self‐made, and the ingredients are 2.5% LC, 10% emulsifiers (4.5% #500 and 5.5% #602), and 87.5% xylene.

### 4.4 MD Simulation

MD simulations were carried out by using the Large‐scale Atomic/Molecular Massively Parallel Simulator (LAMMPS) open‐source software together with the pysimm program. The visualization and image rendering were performed using OVITO software. Additionally, the solubility parameters of the system were calculated using the Materials Studio software with the Conductor‐like Screening Model for Real Solvents (COSMO‐RS) approach. The cohesive energy density was determined through MD simulations. The pore size distributions (PSD) were calculated using Zeo++, employing N_2_ (diameter 1.84 Å) as the probe molecule. Tensile simulations were performed using a quasi‐static method. (The details of the procedure can be found in the Supporting Information)

### 4.5 MD Simulation of pesticide

MD simulations were used to analyze the molecular conformations of LC and Pyr molecules. The structural model of ESO were established using Materials Studio. The simulation algorithm was Forcite‐Dynamics‐Calculation. The simulation was conducted at NVT ensemble at 298 K, while the forcefield was COMPASSII, and charges were forcefield‐assigned. For time parameters, the timestep was 1 fs, the number of steps was 90000, and whole duration was 150 ps.

### 4.6 Molecular Size Calculation of Pesticide

The molecular size of pesticide, was calculated by combining the coordinates of all atoms in the molecule of each kind of atom. The coordinates of atoms were obtained by opening mol file with ChemBio3D.

### 4.7 Characterization of PLOs, LC@PLOs and LC@PLOFs

#### 4.7.1 Performance Measurement of PLOs

IR spectra were recorded using a Fourier transform infrared spectrometer (TENSOR II, Bruker Optics, Germany). A Cryo‐Scanning Electron Microscope (Cryo‐SEM) was used to characterize the dense structure of the material (Hitachi Regulus 8100, Japan). A rheometer was used to evaluate the rheological properties and stress–strain of the material (Anton Paar MCR 302, Austria). DSC was used to evaluate the thermal stability of materials (Netzsch DSC 200 F3, Germany). XRD was used to evaluate the structure of the material (Bruker D8 Advance, Germany). SAXS and WAXS experiments were used to analyze the microstructures of material (Xenocs Xeuss 2.0, France) system, and McSAS software (v1.3.1) was used to simulate and fit the pore size distribution of three kinds of organogels, and sphere model was selected for fitting. The details of the test parameters can be found in the Supporting Information.

#### 4.7.2 Performance Measurement of LC@PLOs

Determination of LC@PLOs pesticide‐loading performance: Accurately weigh 0.1 g of LC@PLOs into a 50 mL centrifuge tube, add 20 mL water, and shake for 30s. After centrifugation, take 0.5 mL supernatant and was diluted, and the impurities were filtered by a 0.22 µm organic filter membrane, and then analyzed by HPLC to obtain the free LC concentration C_1_ that was not supported by PLOs. After taking out the supernatant, add 20 mL acetonitrile to the remaining LC@PLOs after centrifugation, ultrasonication for 30 min, and oscillating extraction for 5 min. After centrifugation, take 0.5 mL supernatant and use acetonitrile and was diluted, and the impurities were filtered by a 0.22 µm organic filter membrane. Then, the LC concentration C_0_ loaded by PLOs was obtained by HPLC with the curve of the standard sample as a reference. The pesticide loading efficiency of LC@PLOs is calculated according to the following formula:

$$LE\;\left(\%\right)=C_{1}/\left(C_{0}+C_{1}\right)\times 100\%$$

SAXS detection of LC@PLOs material is consistent with PLOs, and the radius of gyration (R_g_) is calculated as follows: the R_g_ is estimated by the AUTORG program of ATSAS software (v3.3.0) using the Guinier approximation

$$I\left(q\right)=I\left(0\right)\cdot \exp\left(-\frac{q^{2}R_{g}^{2}}{3}\right)$$

The value of R_g_ is estimated from the best possible linear fit of ln[I(s)] versus s^2^ (Guinier plot), which is valid for sufficiently small scattering vectors.

#### 4.7.3 Performance Measurement of LC@PLOFs

The morphology of the microparticles was observed using a scanning electron microscope (Zeiss Sigma 300, Germany) and a transmission electron microscope (JEM‐1400, Japan). Particle size analysis (90Plus PALS, Brookhaven, America) was conducted to characterize the nanoparticle size and particle distribution. AFM (Bruker Dimension ICON, Germany) was conducted to study the morphology of the flattened state and the adhesion of the microparticles. ImageJ was used to calculate the contact area between the microparticles and the interface. Fluorescence detection of the sample particles was conducted using a high‐resolution laser confocal microscope (LSM 880, Carl Zeiss AG, Germany). The thermal stability of the microparticleswas evaluated using a thermogravimetric analyzer (TGA 55, Waters Corporation, America).

### 4.8 Preparation Characterization of LC@PLOFs

#### 4.8.1 Determination of Pesticide Content in LC@PLOFs

The 0.50 g (± 0.001 g) preparation sample was weighed into a 50 mL centrifuge tube, 30 mL deionized water was added, and centrifuged at 3000 r/min for 5 min after oscillation for 15s. The precipitated part was taken out and dried at 60 C for constant temperature treatment to obtain the dry sample of LC@PLOFs. Weigh the M_0_ (mg) LC@PLOFs dry sample into 50 mL centrifuge tube, add V_0_ (L) acetonitrile, ultrasonic treatment for 30 min, oscillatory extraction for 5 min, centrifuge at 3000 r/min for 5 min, then take 1 mL supernatant and use acetonitrile: The mixture of water (85:15, v/v) was diluted N_0_ times by the supernatant, and the impurities were filtered by 0.22 µm organic filter membrane. Then, the concentration of LC supported by particles C_0_ (mg/L) was obtained by HPLC with the curve of the standard sample as reference. The loading capacity of LC@PLOFs was calculated according to the following formula:

$$\mathrm{LC}\;\left(\%\right)=\frac{C_{0}\times V_{0}\times N_{0}}{M_{0}}\times 100\%$$

#### 4.8.2 Determination of Encapsulation Efficiency of LC@PLOFs

The encapsulation rate (EE, w/w) of LC@PLOFs was determined by using MT 189 (CIPAC) as a reference method. Specific operations are as follows: Weigh 0.10 g (± 0.001 g) LC with M_0_ (mg/L) in a 100 mL glass sample bottle, then add 10 mL deionized water and 50 mL n‐hexane, seal, and place the bottle on a rolling device. After rolling for 15 min at 50 r/min, 1 mL of upper n‐hexane was taken. Impurities were filtered using a 0.22 µm organic filter membrane, and then high‐performance liquid chromatography (HPLC) was performed. The unencapsulated free LC content, M_t_(mg/L), was calculated with reference to the curve of the standard sample. According to the following formula, calculate the encapsulation rate of LC@PLOFs:

$$\mathrm{EE}\;\left(\%\right)=\frac{\left(M_{0}-M_{t}\right)}{M_{0}}\times 100\%$$

#### 4.8.3 Determination of Release Performance of LC@PLOFs

A sample with 0.1 g (±0.001 g) LC content of C_0_ (mg/L) was placed in a 250 mL three‐necked flask, and then 100 mL n‐hexane/ethanol (95:5, v/v) mixture was added to the flask as the release medium. The whole release system was maintained at 25 °C and 300 r/min, and 0.5 mL release medium was taken out at different time points, and 0.5 mL release medium was added at the same time to maintain the volume of the release system. Finally, the extracted release liquid was filtered with a 0.22 µm organic filter membrane, and the impurities were analyzed by HPLC. The concentration C_t_ (mg/L) of LC released to the outside world at different time points was obtained with reference to the curve of the standard sample, and the cumulative release rate curve of LC over time was plotted. The cumulative release rate of LC@PLOFs was calculated according to the following formula:

$$\mathrm{Cumulative}\;\mathrm{release}\;\mathrm{rate}\;\left(\%\right)=\frac{C_{t}}{C_{0}}\times 100\%$$

#### 4.8.4 Determination of UV‐Resistance Performance of LC@PLOFs

First, the LC@PLOFs were diluted to 20 mg/L with deionized water. Then, 1 mL of the LC@PLOFs diluent was evenly applied to a clean slide (2 cm×4 cm) using a pipette gun, and the slide was naturally dried and kept away from light. Then, the slide was placed in a closed space, and the surface was irradiated with UV (0.8 W/m^2^). The slide after UV irradiation was taken out at different intervals, and the residue on the slide was washed with acetonitrile and transferred to a centrifuge tube. After ultrasonic treatment for 30 min, the extraction was carried out by oscillating for 5 min. Finally, the organic phase nitrogen was blown and dried, and the remaining substance was dissolved in 1 mL acetonitrile, and the impurities were filtered by 0.22 µm organic filter membrane. HPLC analysis was carried out, and the concentration of LC remaining on the slide after different UV irradiation times was measured by C_t_ (mg/L) with reference to the curve of the standard sample. C_0_ is the residual concentration of LC on the slide without UV irradiation. The UV degradation curve of LC was plotted. The UV degradation residual rate of LC@PLOFs was calculated according to the following formula:

$$\mathrm{Residual}\;\mathrm{rate}\;\left(\%\right)=\frac{C_{t}}{C_{0}}\times 100\%$$

### 4.9 Evaluation of Foliar Performance of LC@PLOFs

Bioassays after UV Light and Washing. Approximately 0.5 mL of diluent at a concentration of 20 mg L^−1^ was sprayed on round cabbage leaves (diameter: 9 cm). After drying, one group of leaf disks was exposed to UV rays with an average irradiation intensity of 15µW cm^−2^ and removed at 1 and 4 h for bioactivity tests. Another group of leaf disks was washed with water (30°, 20 mL min^−1^) and removed at 4 and 10 min for bioactivity tests. The temperature and the relative humidity were maintained at 25 ± 1 °C and 70 ± 5%, respectively. Larval mortality was evaluated after 24 h. Each experiment was replicated three times.

### 4.10 Bioassay of LC@PLOFs

The leaf‐dip method was developed for the determination of LC@PLOFs insecticidal activity. Each sample was diluted to the gradient diluent with deionized water, and 0.1% Silwet 903 was added to it. Cabbage leaf discs with a diameter of 1 cm were dipped into the sample diluent, removed with a strainer after 20 s, and dried for subsequent use. Two leaf disks and one larva were placed into each hole of 24‐hole cell culture plates. The temperature and relative humidity were maintained at 25 ± 1 C and 70 ± 5%, respectively. Larval mortality was evaluated after 24 h, and LC_50_ was calculated by probit regression analysis. Each experiment was replicated thrice.

The antifungal activity of LC@PLOFs against rice blast was determined by the mycelial growth rate method. Each sample was diluted to the gradient diluent with deionized water, the diluent was evenly mixed with PDA, and transferred to the petri dish. Take out the rice blast fungus on the PDA plate, use the PDA plate without adding samples as a blank control group, and use 9% Pyr EC as a control preparation group. There were three replicates for each treatment. The diameters of mycelial colonies were measured after incubation for 7 days at 25 C by the crisscross method. The inhibitory efficiency of different samples on the mycelia growth of rice blast fungus was calculated, and EC_50_ was calculated.

### 4.11 Greenhouse Efficacy of LC@PLOFs and Pyr@PLOFs

The sample was diluted with deionized water to 20 mg/L diluent according to the active composition, and 0.01% of the organosilicon additive was added. Spray 30 mL of sample diluent evenly onto the kale plants. Each sample was treated with two cabbage plants, and the experiment was divided into two groups. After the first group of liquid medicine was dried, the leaves were taken for use at different time points (0, 1, 3, and 5 days). In the second group, a small spray device was used to spray deionized water evenly on the surface of the blades at a flow rate of 15 mL/min at an Angle of 45° on the top 20 cm before taking the blades, and the water consumption was 50 mL to simulate the flushing behavior of rainwater on the blade surface. The leaves were treated as leaf cakes with a diameter of 1 cm. One larva and two cabbage leaf cakes were placed in each hole of the 20‐hole cell culture plate and placed in the larva feeding environment. After 24 h, the death of larva was observed and recorded, and the corrected mortality of larva was calculated. Deionized water containing 0.01% silicone was used as blank control group, and high efficiency LC EC was used as control group. The experiment was repeated three times.

According to NY/T 1156.8‐2007, the greenhouse test of rice blast control with Pyr@PLOFs was carried out. First, spore suspension was prepared, blast blast cake was inoculated into SDC medium, and after 3 days of induction under black light, mycelia was scraped off, and spores were collected with deionized water for further induction for 3 days, which was used as spore suspension for reserve. Then the sample was diluted to 30 mg/L diluent with deionized water according to the active composition, and 0.01% of the organosilicon additive was added. The 15 mL sample diluent was evenly sprayed onto the rice plants and left for natural drying. The deionized water containing 0.01% silicone was used as blank control, and Pyr EC was used as control. After 24 h, the spore suspension was sprayed and inoculated on the rice plants, and the plants were placed in a humidor with a relative humidity of more than 95% and a temperature of 25°C. The plants were cultured without light for 24 h, and then alternated between light and dark for 12 h. According to the method in the standard, the disease grade was investigated and calculated, and the disease index and prevention effect were calculated. The field efficacy of LC@PLOFs and Pyr@PLOFs are described in the Supporting Information.

### 4.12 Safety Evaluation of LC@PLOFs

Toxicity Assessments of Adult Zebrafish. Adult zebrafish (*D.rerio*, n = 15) were randomly selected and distributed into a 10L tank with 5L sample gradient diluent. Clean water served as the control treatment. During the experiment, the entire sample diluent was renewed every 24 h to maintain the pesticide concentration of LC and water quality. The number of dead fish in each tank was recorded after 96 h, and the mortality and LC_50_ were obtained by probit regression analysis. The judgment standard of death status was no breathing or movement by touching the tail. All procedures involving fish were reviewed and approved by the Animal Ethics Committee of Anhui Agricultural University and conducted in compliance with Chinese legislation (Approval No. KJLL2025044).

The activities of chlorophyll and glucose were detected by corresponding assay kits according to the manufacturer's protocol (Jianglai Biotech, Shanghai, China).

### 4.13 Morphology Characterization of LC@PLOFs After Hydrolysis

An appropriate amount of dry sample was ultrasonic dispersed in deionized water as a sample diluent, and the sample diluent was evenly applied to the silicon wafer and the slide, respectively, and dried for characterization. The silicon wafers and slides coated with different particle samples were put into a 50 mL centrifuge tube, and an appropriate amount of deionized water was added, so that the LC@PLOFs were completely immersed in the deionized water and stored in a sealed condition at room temperature. After 40 days, the silicon wafers and slides were removed, and after natural drying, the morphology characteristics of the hydrolyzed LC@PLOFs were observed by an imaging microscope and a scanning electron microscope.

### 4.14 Statistical Analysis

The data from the bioassays, field evaluation, release, UV, and washout resistance were statistically analyzed using SPSS software (version 24) and displayed as the means and the standard error (SE) by Tukey's multiple range test (*p* < 0.05).

## Conflicts of Interest

The authors declare no conflict of interest.

## Supporting information




**Supporting file 1**: advs74129‐sup‐0001‐SuppMat.docx.

**Supporting file 2**: advs74129‐sup‐0002‐VideoS1.mp4.

**Supporting file 3**: advs74129‐sup‐0003‐VideoS2.mp4.

## Data Availability

The data that support the findings of this study are available from the corresponding author upon reasonable request.
