# Supplementary material for: Tailored Organogel Systems for Optimized Pesticide Delivery: Mechanistic Insights and Agricultural Applications
Source: Adv Sci (Weinh). 2026 Jan 28;13(20):e19352. doi: 10.1002/advs.202519352 (PMC13067833; doi:10.1002/advs.202519352)
Supplement: Supplementary file 1 — Supporting file 1: advs74129‐sup‐0001‐SuppMat.docx. [file ADVS-13-e19352-s002.docx]

Supporting Information

**Tailored Organogel Systems for Optimized Pesticide Delivery: Mechanistic Insights and Agricultural Applications**

*Yue Wang, SiYu Xv, Chao Wu, Nan Li, GuoPeng Teng, XuePing Huang,*

*Jian Luo^*^*

Y. Wang, S. Xv, C. Wu, N. Li, Prof. J. Luo

School of Plant Protection, Anhui Agricultural University, Hefei, Anhui Province 230036, P.R. China

G. Teng

School of Chemistry and Materials Science, University of Science and Technology of China, Hefei 230026, Anhui, China

X. Huang

Institute of Plant Protection and Agro-Product Safety, Anhui Academy of Agricultural Sciences, Hefei, Anhui 230001, P. R. China.

*Corresponding Author

E-mail: [luojian@ahau.edu.cn](mailto:luojian@ahau.edu.cn)

This file includes: Experimental Section, Figures S1-S15 and Tables S1-S3.

Other Supporting Materials for this manuscript include the following: Movies S1-S2.

1. **Experimental Section**

**1.1 Insect Source**

**Feed formulation:** Add 50 g of agar powder into 1 L of water, heat and dissolve, add 100 g of malt powder, 150 g of corn flour, and 50 g of soybean powder, stir well. Then add sorbic acid and cool to 50 °C. Finally, add formaldehyde and vitamin C and cool to 4°C.

**1.2 Molecular Dynamics (MD) Simulation**

The simulation system was constructed using the PACKMOL package by stacking monomer molecules into the box.^[1-3]^ The monomer molecules was modelled by the pcff force field and the reaction atom in the monomer molecule was set as R1 and R2 to complete the cross-linking reactio.^[4,5]^ The models were used by a periodic boundary condition and set the cutoff radius as 1 nm, and the Ewald method was used to describe the Long range interaction force. Before the MD simulation, the cross-linking reaction was performed to form crosslinked polymer. In detail, the close contacts distance of the crosslinking reaction is set to 4.5 Å to 8.5 Å, before the cross-linking reaction simulation at every contacts distance, the system firstly annealed under the circulating temperatures ranging from 300-500 K under the NPT (P = 101 KPa) ensemble for 20 ps. The MD simulation of the cross-linking reaction was performed under the NVT ensemble (T = 300 K) for 50 ps to obtain the final crosslink polymer structures. The all initial models were firstly energy-minimized by using steepest descent algorithm, and the temperature and pressure were controlled by Nose-Hoover thermostat and Parrinello-Rahman barostat, respectively. The density distributions of reaction atom in the monomer molecule was also calculated.

Additionally, the solubility parameters of the system were calculated using the Materials Studio software with the Conductor-like Screening Model for Real Solvents (COSMO-RS) approach. Initially, the target density systems were constructed and geometrically optimized within Materials Studio. The cohesive energy density was determined through molecular dynamics simulations.

The nonbonded interaction contains van der Waals (vdW) and electrostatic interaction, which is described by the Equation 1 and Equation 2, respectively.

$E_{LJ}\left( r_{ij} \right)=4\varepsilon_{ij}\left( \left( \frac{\text{σ}_{ij}}{r_{ij}} \right)^{12}-\left( \frac{\text{σ}_{ij}}{r_{ij}} \right)^{6} \right)$ (1)

$E_{c}\left( r_{ij} \right)=\frac{q_{i}q_{j}}{4\pi\varepsilon_{o}\varepsilon_{r}r_{ij}}$ (2)

In the equation, $q_{i}$、$q_{j}$ are atomic charge, $r_{ij}$is the distance between atoms, $\text{σ}$ is the atomic diameter, $\varepsilon$ is the atomic energy parameter.

For different kinds of atoms, the geometric mix rules were adopted for vdW interactions, which is following the Equation 3. The cutoff distance of vdW and electronic interactions was set to 1.2 nm, and the PPPM method was employed to calculate long-range electrostatic interactions.

$\sigma_{ij}=\frac{1}{2}\left( \sigma_{ii}+\sigma_{jj} \right);\varepsilon_{ij}=\left( \varepsilon_{ii}*\varepsilon_{jj} \right)^{\frac{1}{2}}$ (3)

Tensile simulations were performed using a quasi-static method, generating successive configurations following deformation and subsequent minimization of potential energy.^[6]^ To model the tensile, two infinite planar force fields representing the nano-indenter and the substrate were positioned at the top and bottom surfaces of the sample, following the approach introduced by Issa and collaborators.^[7]^ Each force field was defined as a repulsive harmonic potential, with its normal aligned along the tensile axis, characterized by a spring constant 𝐾 = 100 eV/Å. During the tensile process, the indenter was iteratively displaced along the z-axis (the direction of compression) with a stepwise displacement corresponding to an engineering strain increment of 𝛥𝜀𝑧𝑧 = 0.1%. The zero strain reference state was established at the onset of contact homogeneity between the surfaces of the nano-cube and the indenters, typically preceded by a transient accommodation stage characterized by partial contact. The tensile axis z was fixed and initially aligned with the (001) direction of the nanocrystal.^[8]^

**1.3 Performance measurement of PLOs**

The dynamic rheological behaviors of the samples were examined using a rheometer equipped with a flat parallel plate (15 mm in diameter). All characterizations were performed within the linear viscoelastic region at 25°C. The time sweep test was carried out at 2% strain and 1 rad/s frequency over a time range from 0 to 300 s. Measurement of yield stress in rotating mode at 25 °C.

The thermal behavior of the gels was measured by using DSC equipped with a cooling system. In N^2^ atmosphere, the initial equilibrium temperature is -100 ℃, and the temperature rises to 200℃ at the rate of 10 ℃/min. Then the temperature is lowered to 30 ℃ at 10 ℃/min.

X-ray diffractometry (XRD) measurements were performed with Cu anode (CuKa, l = 1.54 Å), where XRD patterns were collected in a range from 5°to 90°in 2q by a step-scan mode withthe scanning speed is 2°/min.

SAXS and WAXS experiments were conducted with an Xenocs Xeuss 2.0 system. X-Ray wavelength: 1.54189 Å. Detector: Pilatus 3R 300 K. Single pixel size: 172 μm. Sample detector distance: 1188 mm (SAXS) and 93.6 mm (WAXS). Exposure time: 300 s. Experimental environment: room temperature vacuum. Collimation mode: High throughput mode. The data was analyzed in the XSACT software and corrected for background scattering from air and from the sample holder. The lengths of the scattering vector q were 0.007375 Å^-1^ to 0.419772 Å^-1^ for SAXS, and were 0.071473 Å^-1^ to 2.349391 Å^-1^ for WAXS.

The pore size distributions were conducted from MCSAS (v1.3.1) by a Monte Carlo rejection sampling technique from the scattering data. At the core of the approach is a set of independent, non-interacting scatterers, each of which is modeled according to a sphere scatterer model. The process begins with a set of non-interacting scatterers, and calculated as the weighted sum of the scattering patterns from all scatterers in the set. After 10 iterations of the Monte Carlo procedure until the convergence criterion of χ² ≤ 1，the agreement between the model and the measured data was accepted^[9,10]^.

**1.4 Field efficacy of LC@PLOFs and Pyr@PLOFs:**

Field Efficacy Against Cercospora Sojina. The field trial was conducted in the soybean planting area of Pangnali Village, Shanglaozhuang Township, Dongping County, Tai'an City, Shandong Province, where soybean gray leaf spot had already occurred extensively to verify the efficacy of the treatment. The plots were set up as 5-row zones, each row measuring 5 meters in length, with a 1.0-meter-wide isolation belt between plots. The samples were then diluted with farmers' domestic water to 60 mg/L based on the active ingredient. To enhance the wetting and spreading effect of the diluted solution on leaves, 0.01% silicone adjuvant was added to the solution. Each plot was sprayed with 5 liters of the solution using a sprayer to ensure that the soybean plants were completely wet. Deionized water containing 0.01% silicone was used as the blank control, and Pyr EC served as the chemical control. Three replicates were set up in each plot. Twenty-eight days after application, the disease grade of all leaves on 10 randomly selected soybean plants in each plot was recorded, and the disease index and control effect were calculated accordingly. Thirty days after application, a yield test was conducted, where 10 soybean plants were randomly selected from each plot to record plant status, and the fresh and dry weight of pods per plant were measured.

Field Efficacy Against Pieris rapae. The field trial was conducted in the planting area of Liangzhuang Village, Fangcun Town, Daiyue District, Tai'an City, Shandong Province. The experiment was chosen to start two weeks after planting. Each plot had an area of 15 m^2^ with at least 50 plants. A 1.0 m wide isolation belt was set up between plots. Before applying pesticides, the baseline population of insects was investigated. The samples were diluted with tap water to 20 mg/L according to the active ingredient. To improve the wetting and spreading effect of the diluted solution on leaves, 0.01% silicone was added as a wetting agent. Each plot was sprayed with 2 L of the solution using a sprayer to ensure that the leaves were completely wet. Deionized water containing 0.01% silicone was used as the control group, and LCEC as the control treatment group. Three replicates were set up in each plot. The number of insects in each plot was counted and recorded on the 1st and 7th day after spraying. The insect reduction rate and efficacy were calculated. The weather and rainfall during the control period were recorded.

1. **Supp****orting Figures and Tables**


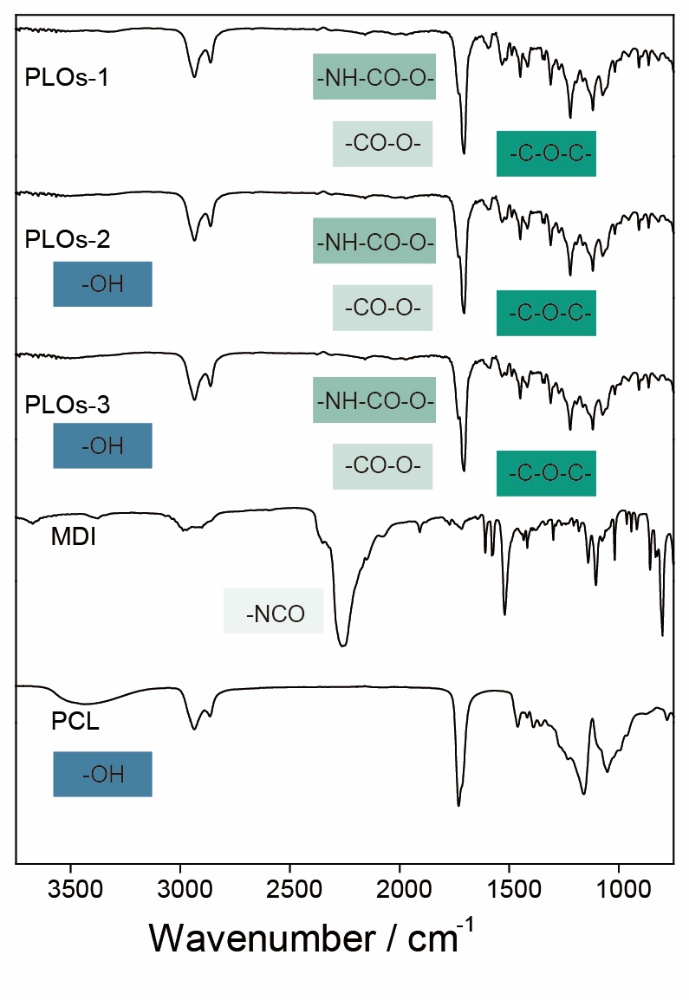


**Figure S1.** IR spectra images of PLOs samples.


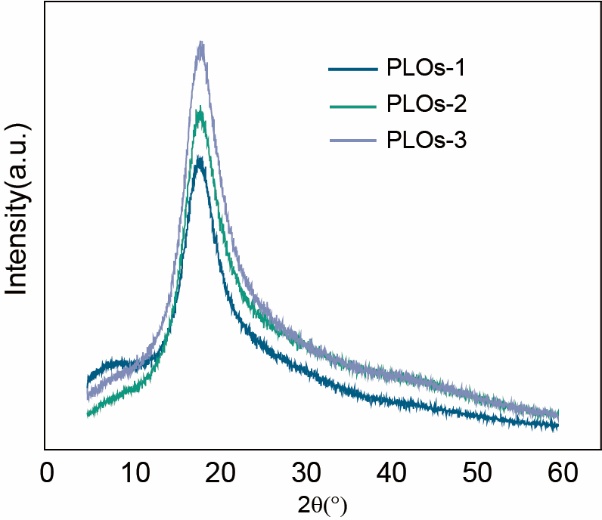


**Figure S2.** XRD images of PLOs samples.


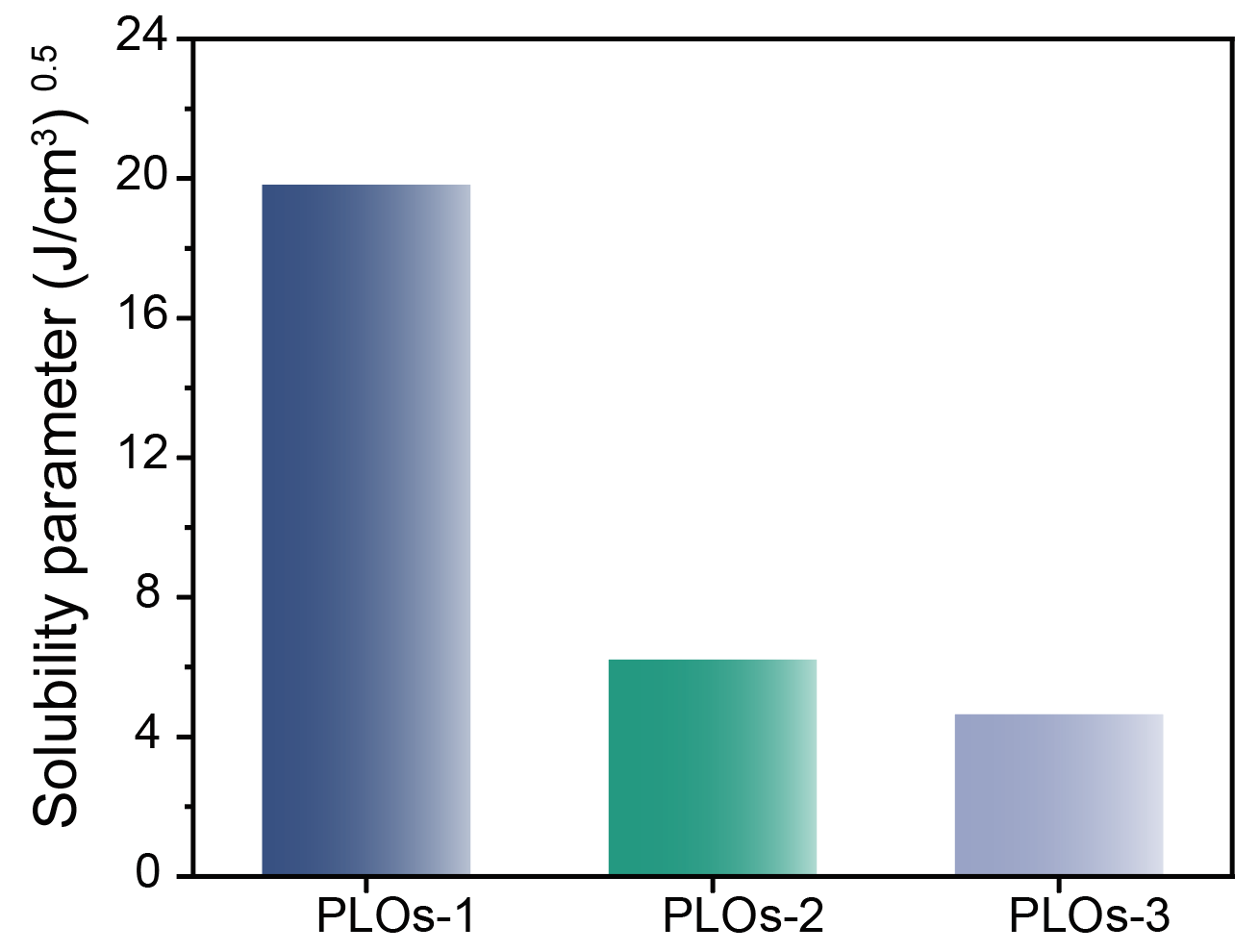


**Figure S3.** Solubility parameter of PLOs samples


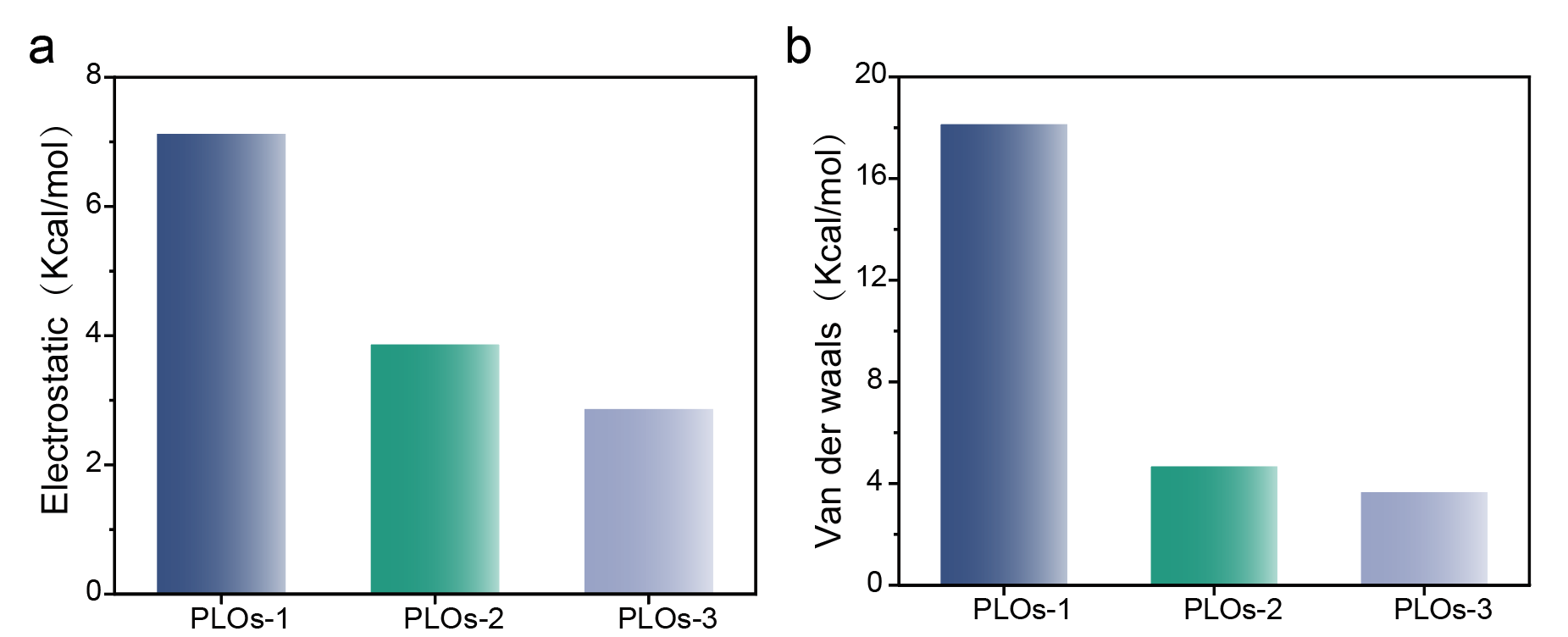


**Figure S4.** Intermolecular forces of PLOs samples: (a)Electrostatic of PLOs samples. (b) Van der Waals of PLOs samples.


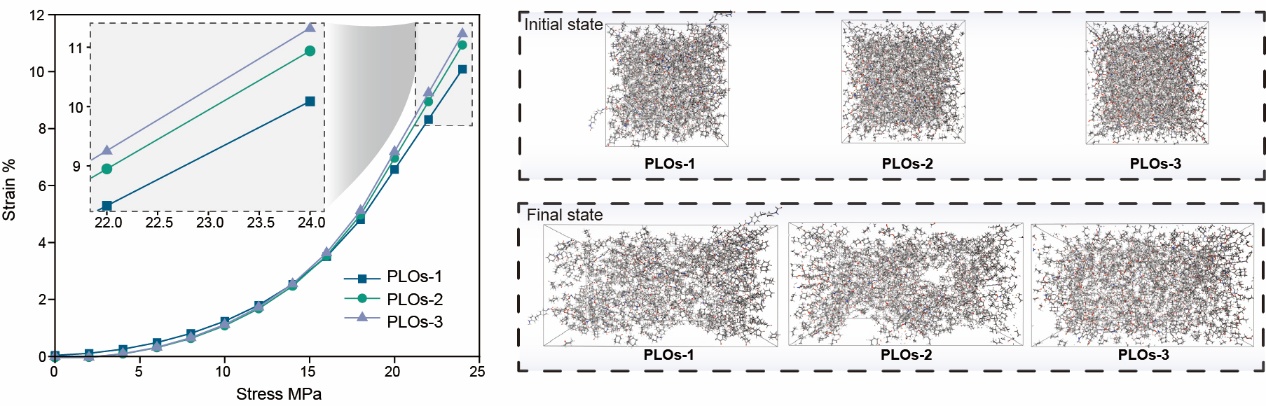


**Figure S5.** Simulated tensile stress-strain curves and images of PLOs samples.


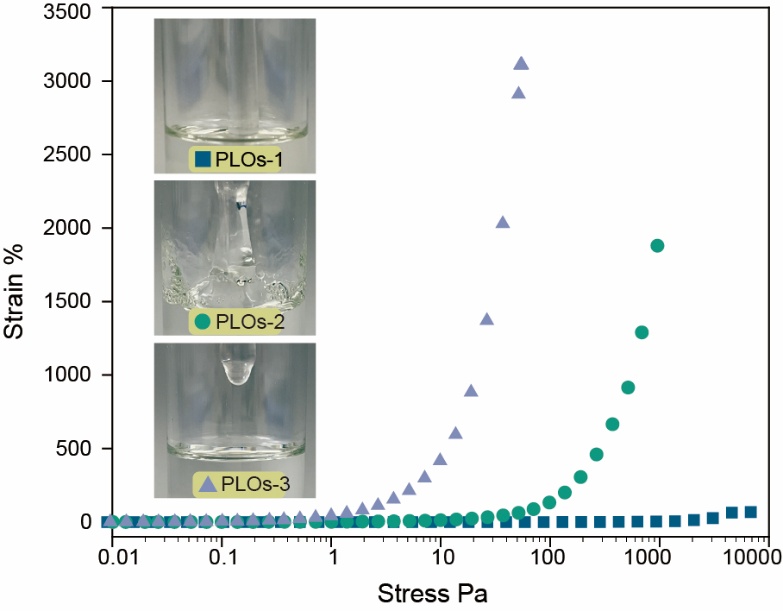


**Figure S6.** Rotational stress-strain curves of PLOs samples.


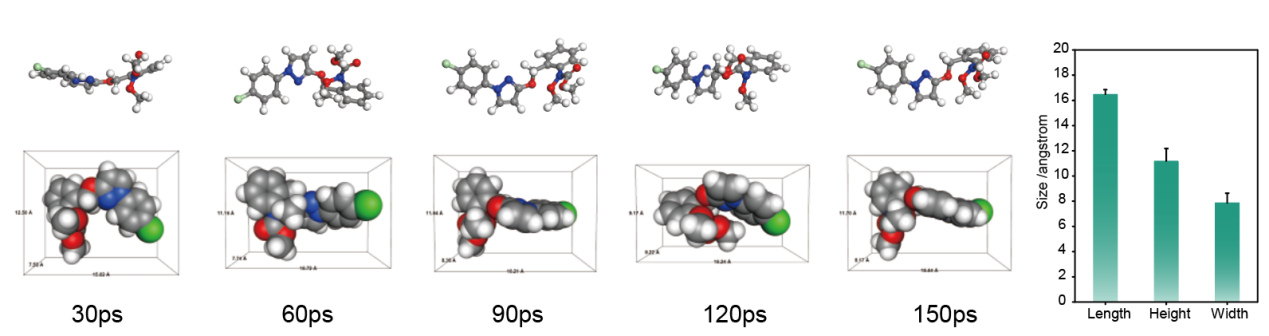


**Figure S7.** Molecular Dynamics (MD) Simulation of Pyr


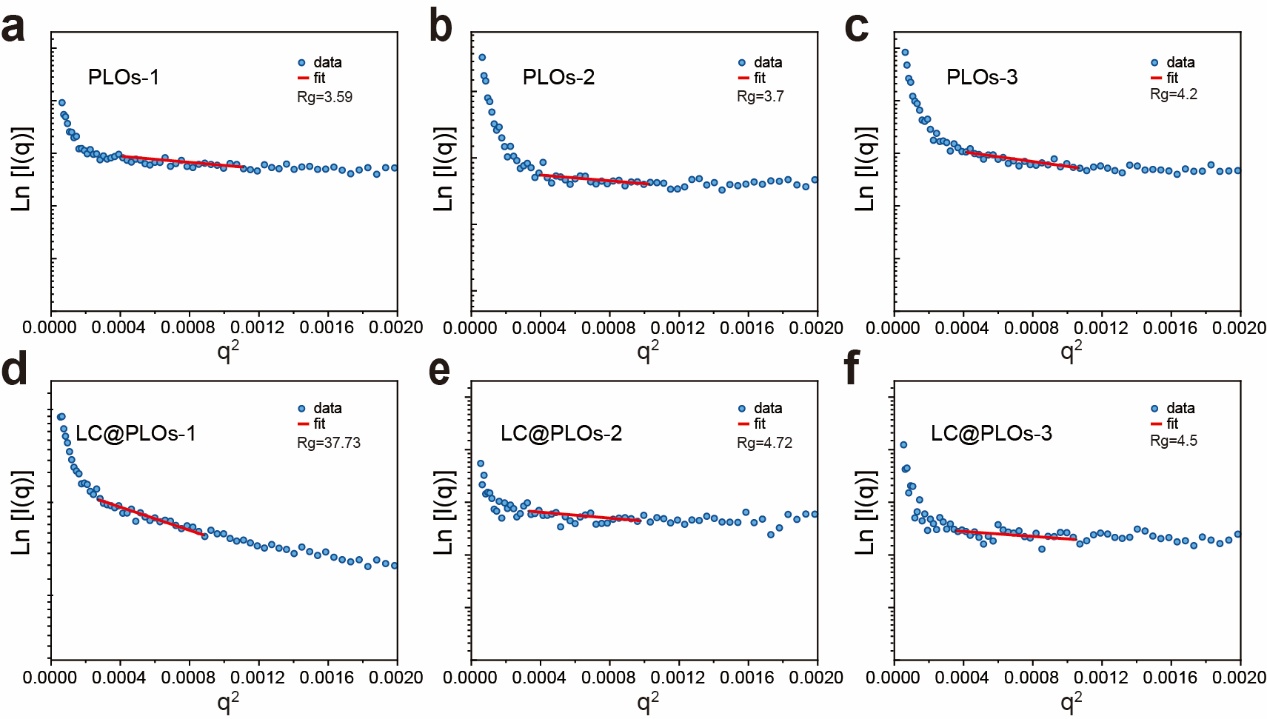


**Figure S8.** Radius of gyration for PLOs and LC@PLOs samples.


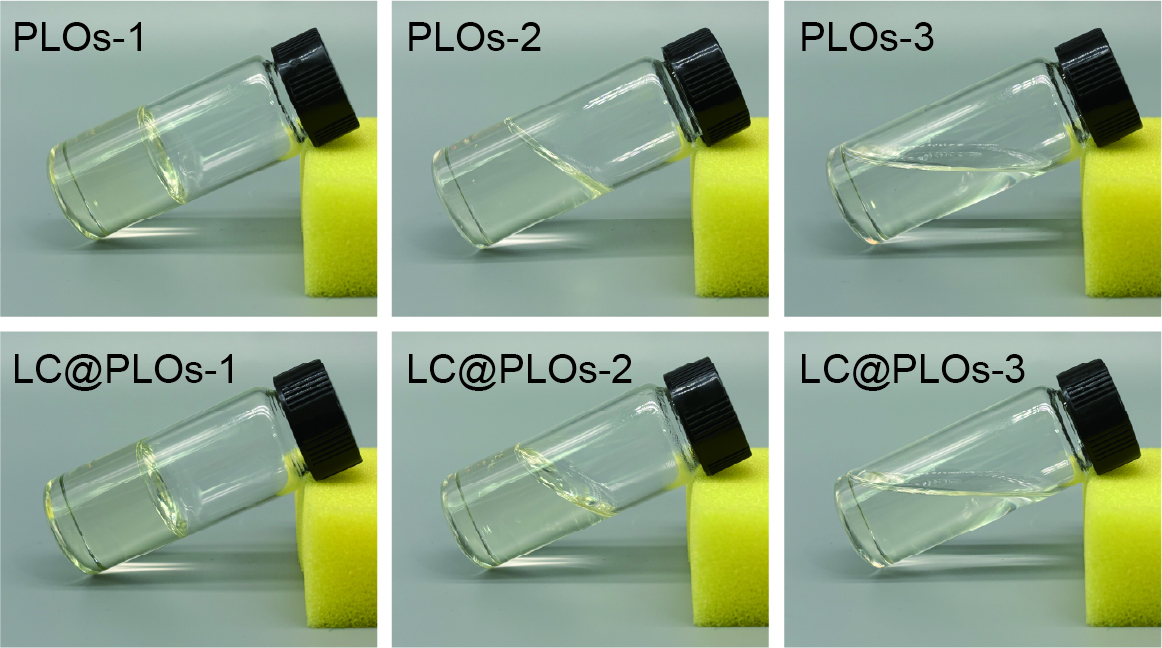


**Figure S9** Status images for PLOs and LC@PLOs samples.


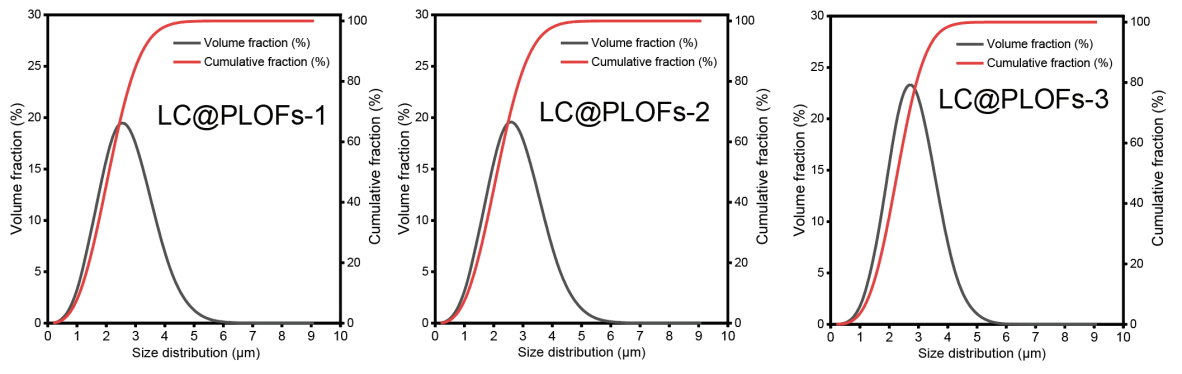


**Figure S10.** Size distribution of LC@PLOFs samples.


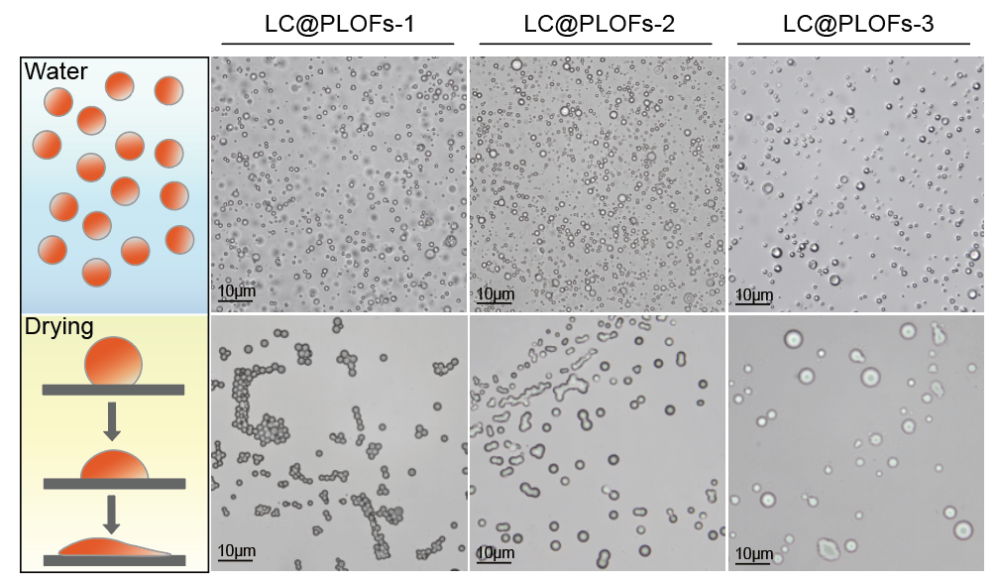


**Figure S11.** Morphological characteristics of LC@PLOFs samples before and after water evaporation


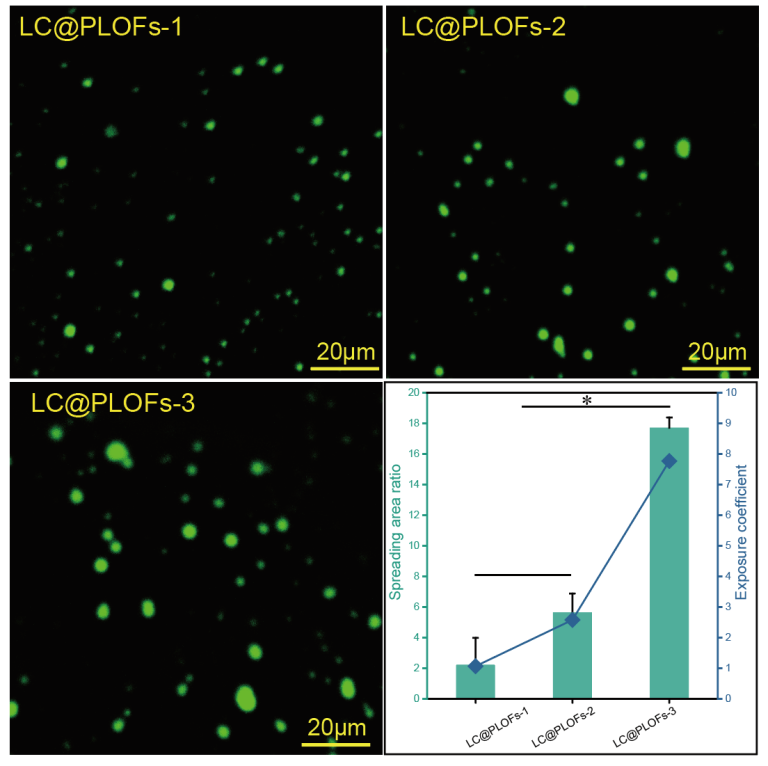


**Figure S12.** Distribution state and spreading area ratio of LC@PLOFs samples.


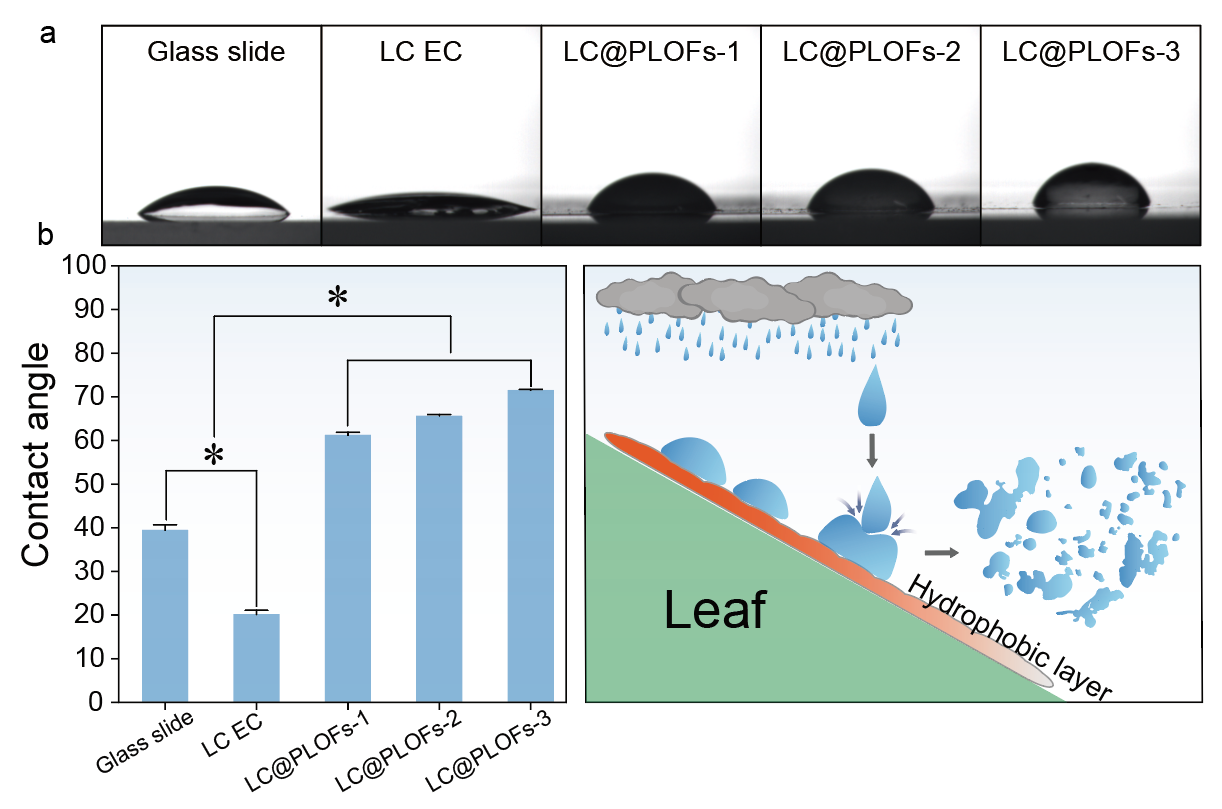


**Figure S13** Wettability (a) and contact angle (b) of water droplets on the coating of LC@PLOFs.

LC EC is mixed with the solvent to form an emulsion layer under the action of a surfactant, resulting in a state of easy wetting. However, the wetting state of water droplets on the LC@PLOFs coating is significantly reduced, which proves that the LC@PLOFs coating can improve the hydrophobicity of the surface of the glass slide, and can play a better protective effect on the rain impact than emulsion preparation, thereby improving the washing resistance of the leaf surface. The contact angle of LC@PLOFs water droplets was more than 3 times higher than that of LCEC (figure S13b.). After the application of LC@PLOFs, with the evaporation of water on the leaf surface, the LC@PLOFs in the liquid can provide a new polymer coating for the leaf application site that loses the protection of the wax layer, thereby reducing the pesticide loss caused by the continuous erosion of rainwater.


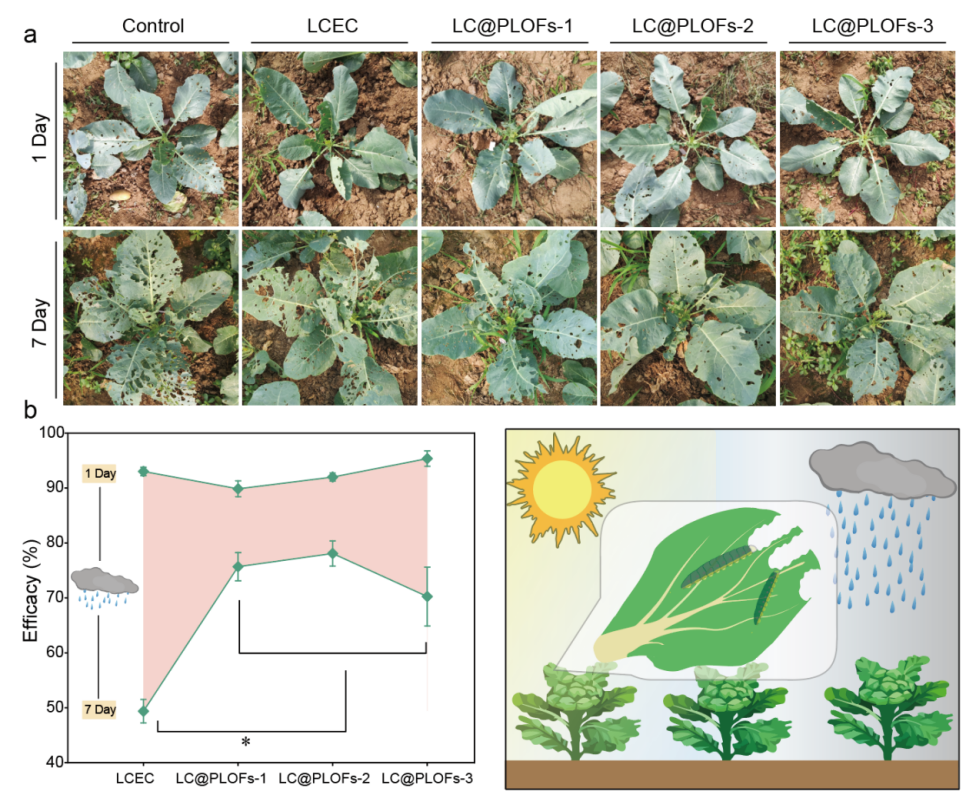


**Figure S14** Insecticidal Activity of LC@PLOFs After Washing in Field Conditions. Growth status of cabbage(a); Field efficacy(b). All experiments were performed three times, and error bars represent standard deviation (SD). *, P < 0.05.

The field efficacy of LC@PLOFs was shown in figure S14. The control results within the first seven days are shown in figure S14a. On the first day post-application, all treatments exhibited strong initial efficacy, suggesting that excessive pesticide coating and limited exposure in the field may reduce the immediate effectiveness of insecticides. By the seventh day, the efficacy of the LC EC treatment had declined to 49.4% due to the combined effects of intense sunlight, heavy rainfall, and reinfestation by external insect populations. In contrast, the overall efficacy of LC@PLOFs remained above 70%, demonstrating a significant advantage over LCEC (Figure S14b). Notably, the level of leaf protection closely aligned with the efficacy data, with LC@PLOFs treatments significantly reducing leaf damage. Among them, the LC@PLOF-3 treatment exhibited the best leaf condition.


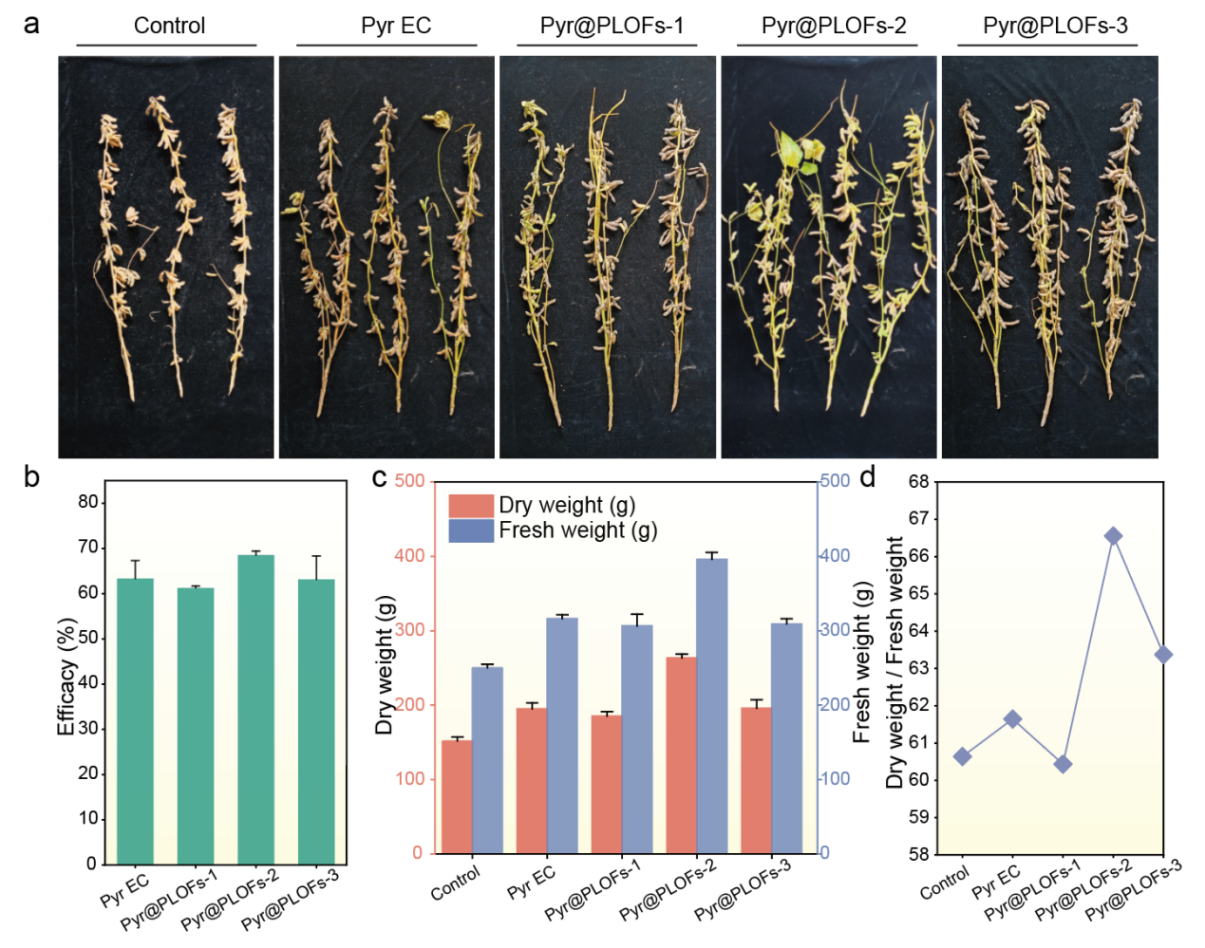


**Figure S15** Insecticidal Efficacy of Pyr@PLOFs Against Grey Speck of Soybean. (a) soybean status; (b) Efficacy; (c) Fresh and dry weight of pods; (d) Dry/fresh weight. All experiments were performed three times, and error bars represent standard deviation (SD). *, P < 0.05.

At the same time, the field efficacy of Pyr@PLOFs on *cercospora sojina* was verified. As shown in figure S14b, the efficacy of each treatment was greater than 58%. In the control group (Figure S15a), soybean plants exhibited the most severe symptoms of grey speck disease. Extensive leaf shedding during the infection period resulted in stunted and desiccated plants at the late harvest stage, with sparsely distributed pods, most of which appeared shriveled. However, the plants and pods treated by Pyr@PLOFs-2 were healthier and more tender, and there were still some leaves on them, which had better protection effect on plants. The pod dry weight and fresh weight data of soybean showed that the pod yield correlated positively with the treatment efficacy against *cercospora sojina* (figure S15c). Moreover, from the ratio of dry weight to fresh weight (figure S15d), the control treatment was 60.6%. The results of Pyr EC treatment and Pyr@PLOF-1, Pyr@PLOF-2 and Pyr@PLOF-3 treatment were 61.6%, 60.4%, 66.5% and 63.4%, respectively. Notably, a higher efficacy rate corresponded to an increased dry-to-fresh weight ratio, indicating improved yield protection.

**Table S1.** Composition ratio of PLOs samples in different flexible states

| Sample | PCL  (g) | MDI  (g) | Cyclohexanonel  (g) |
| --- | --- | --- | --- |
| PLOs-1 | 1.5 | 1.5 | 10 |
| PLOs-2 | 3 | 1.5 | 10 |
| PLOs-3 | 4.5 | 1.5 | 10 |

**Table S2.** Composition ratio of LC@PLOs samples in different flexible states

| Sample | PCL  (g) | MDI  (g) | Pesticide  (g) |
| --- | --- | --- | --- |
| LC@PLOs-1 | 1.5 | 1.5 | 2.6 |
| LC@PLOs-2 | 3 | 1.5 | 2.6 |
| LC@PLOs-3 | 4.5 | 1.5 | 2.6 |

**Table S3.** Composition ratio of LC@PLOFs and Pyr@PLOFs samples in different flexible states

| Sample | PCL  (g) | MDI  (g) | Pesticide  (g) |
| --- | --- | --- | --- |
| LC@PLOFs-1 | 2.5 | 2.5 | 2.6 |
| LC@PLOFs-2 | 3.33 | 1.67 | 2.6 |
| LC@PLOFs-3 | 3.75 | 1.25 | 2.6 |
| Pyr@PLOFs-1 | 3.5 | 3.5 | 9.28 |
| Pyr@PLOFs-2 | 4.66 | 2.34 | 9.28 |
| Pyr@PLOFs-3 | 5.25 | 1.75 | 9.28 |

1. **List of the Movies**

**Video S1.** Video of macroscopic state of PLOs samples.

**Video S2.** Video of hydrophobic properties of PLOs samples.

**Reference**

[1] Plimpton, S. Fast Parallel Algorithms for Short-Range Molecular Dynamics. J. Comput. Phys. 117, 1−19 (1995). https://doi.org/10.1006/jcph.1995.1039

[2] Nayir, N., Duin, A. & Erkoc, S. Development of a ReaxFF Reactive Force Field for Interstitial Oxygen in Germanium and Its Application to GeO2/Ge Interfaces. J. Phys. Chem. C. 123, 1208-1218 (2018). https://pubs.acs.org/doi/pdf/10.1021/acs.jpcc.8b08862

[3] Martínez L, Andrade R, Birgin E.G., & Martínez J.M. PACKMOL: a package for building initial configurations for molecular dynamics simulations. J. Comput Chem. 30, 2157-2164(2009). https://doi.org/10.1002/jcc.21224

[4] Berendsen, H. J. C., Grigera, J. R., & Straatsma, T. P. The Missing Term in Effective Pair Potentials. J. Phys. Chem., 91, 6269-6271(1987). https://doi.org/10.1021/j100308a038

[5] Ryckaert, J. P., Ciccotti, G., & Berendsen, H. J. C. Numerical Integration of a System with Constraints: Of the Cartesian Equations of Motion Molecular Dynamics of n-Alkanes. J. Comput. Phys. 23, 327−341(1977). <https://doi.org/10.1016/0021-9991(77)90098-5>

[6] Allen, M. P., & Tildesley, D. J. Computer Simulation of Liquids. Oxford University Press, 1987. https://doi.org/10.2307/2938686

[7] Alexander Stukowski. Visualization and analysis of atomistic simulation data with OVITO–the Open Visualization Tool. Modelling Simul. Mater. Sci. Eng. 18, 015012 (2010). <https://doi.org/10.1088/0965-0393/18/1/015012>

[8] Honeycutt, J. D. Molecular dynamics study of melting and freezing of small Lennard-Jones clusters. J. Phys. Chem. 91, 4950-4963(1987). <https://doi.org/10.1021/j100303a014>

[9] Şenel, İ. G., Gürüz, A. G., Yücel, H., & Kandas, A. W. Characterization of pore structure of Turkish coals. [ENERG FUEL](https://www.ablesci.com/journal/detail?id=DYQ9vD). 15, 331-338 (2001). https://doi.org/10.1021/ef000081k

[10] Bressler I., Pauw B. R. & Thunemann A. F. McSAS: software for the retrieval of model parameter distributions from scattering patterns. J. Appl. Cryst. 48, 962-969 (2015). https://doi.org/10.1107/s1600576715007347
